# DNA barcoding and a precise morphological comparison revealed a cryptic species in the *Nippolachnus piri* complex (Hemiptera: Aphididae: Lachninae)

**DOI:** 10.1038/s41598-018-27218-2

**Published:** 2018-06-13

**Authors:** Mariusz Kanturski, Yerim Lee, Jinyeong Choi, Seunghwan Lee

**Affiliations:** 1 Department of Zoology, Faculty of Biology and Environmental Protection, University of Silesia in Katowice, Bankowa 9, 40-007 Katowice, Poland; 2 Laboratory of Insect Biosystematics, Department of Agricultural Biotechnology, Research Institute of Agriculture and Life Sciences, Seoul National University, Seoul, 08826 Republic of Korea

## Abstract

*Nippolachnus* is a small Palaearctic-Oriental genus of very characteristic aphids that live on the leaves of woody Rosaceae. One species, *N*. *piri*, has hitherto been regarded to be widely distributed and relatively polyphagous. Members of this genus are considered to be easy to recognize due to the absence of the ocular tubercle and triommatidia on the head. We conducted research on the morphology and generic characters of *Nippolachnus piri* complex using scanning electron microscopy (for the first time) and DNA barcoding. We analyzed *N*. *piri* populations on *Pyrus* and other plants (*Eriobotrya*, *Rhaphiolepis* and *Sorbus*) in Japan and the Republic of Korea. Specifically, a high genetic divergence value was found between the *N*. *piri* populations associated with different host plants. SEM investigation of the head capsule revealed that a triommatidium is present under the compound eye, despite their lack of an ocular tubercle. We propose *Nippolachnus micromeli* Shinji, 1924 **stat**. nov. as a cryptic species in the *N*. *piri* complex based on a morphological comparison, DNA barcoding and different host-plant associations. Illustrations and descriptions of studied species are given. Morphological keys to the apterae and alatae of all known species of the genus *Nippolachnus* are also provided.

## Introduction

Aphids are a species-rich group of taxa that include many economically important pests^1^. Aphids that belong to the subfamily Lachninae are one of the most interesting groups among phytophagous insects due to their ability to feed on both the leafy and woody parts of both coniferous and deciduous plants^2^. The subfamily has been widely researched in terms of their phylogeny and speciation^3–6^. On the other hand, there are very few studies on the morphology and modern systematics at a higher taxonomic level or analyses of the species complexes^7–9^

The Lachninae genus *Nippolachnus* from the tribe Tuberolachnini is known from Southeast Asia^10–12^. Matsumura^13^, described the first species – *N*. *piri* from specimens that had been collected from *Pyrus* and simultaneously created a new genus. In addition, van der Goot^14^ described one species of this genus that had been collected in the Himalayas – *N*. *himalayensis*, which due to some of its unusual morphological characters was placed in the genus *Lachnus* for a long time. Shinji^15^ described a poorly known *Nippolachnus* species, *N*. *micromeli*, from *Sorbus alnifolia* (=*Micromeles alnifolia*) from Japan. Like *N*. *himalayensis*, another species, *N*. *bengalensis*, which was described from West Bengal^16^, was treated as being rare and endemic to Indian mountain areas. Based on this information, *N*. *piri* has been treated as a very common and widely distributed species that occurs in China, Japan, Korea and Taiwan and is regarded as being polyphagous on various plants: *Eriobotrya*, *Betula*, *Pyrus*, *Rhaphiolepis*, *Sorbus* and *Ulmus*^10,12,17^. *Nippolachnus micromeli* has been synonymised with *N*. piri by Tao^18^ (page 44), unfortunately without an appropriate comment and this was consistently upheld by other authors^19^, most probably because it is poorly known and due to Shinji’s brief description. It is likely that *N*. *piri* was regarded as being a polyphagous species with a wide range similar to that of some other well-known polyphagous lachnids, e.g. *Trama troglodytes*, *Longistigma caryae*, *Lachnus tropicalis* or the recently discovered *Stomaphis wojciechowskii*^6,17,20^. Despite many studies of Lachninae in Asia, little was known about the morphology and biology of *Nippolachnus* besides observations of the oviposition of *N*. *piri* on *Rhaphiolepis umbellata* that were made by Ôtake^21,22^, who regarded this plant as the winter host for *N*. *piri*. However, *N*. *piri* is apparently anholocyclic in India (without a sexual generation) and has only been recorded from *Pyrus*^23^.

Species identification of Lachninae can be difficult due to their morphological ambiguity^24^. DNA barcodes have frequently been attempted in order to get a more reliable species identification and recognition of cryptic species in other groups of aphids^25–31^. For a more rapid and precise species diversity estimation, this method of discovering diversity has been extensively adopted for various groups of taxa^32–34^.

In aphids, different host-plant associations can provide critical evidence that can be used to detect cryptic species^35,36^. To clarify whether *N*. *piri* is indeed a polyphagous aphid or a species complex, we performed combined molecular and morphological analyses for populations that had putatively been assigned to *N*. *piri* on four host-plants (*Eriobotrya*, *Pyrus*, *Rhaphiolepis* and *Sorbus*). As a result of these studies, we propose that the populations on *Rhaphiolepis* and *Sorbus*, which were hitherto identified as *N*. *piri*, are a distinct, cryptic species that is different from the original *N*. *piri*.

## Results

### Genetic variation of the *Nippolachnus piri* species complex

**Figure 1:**
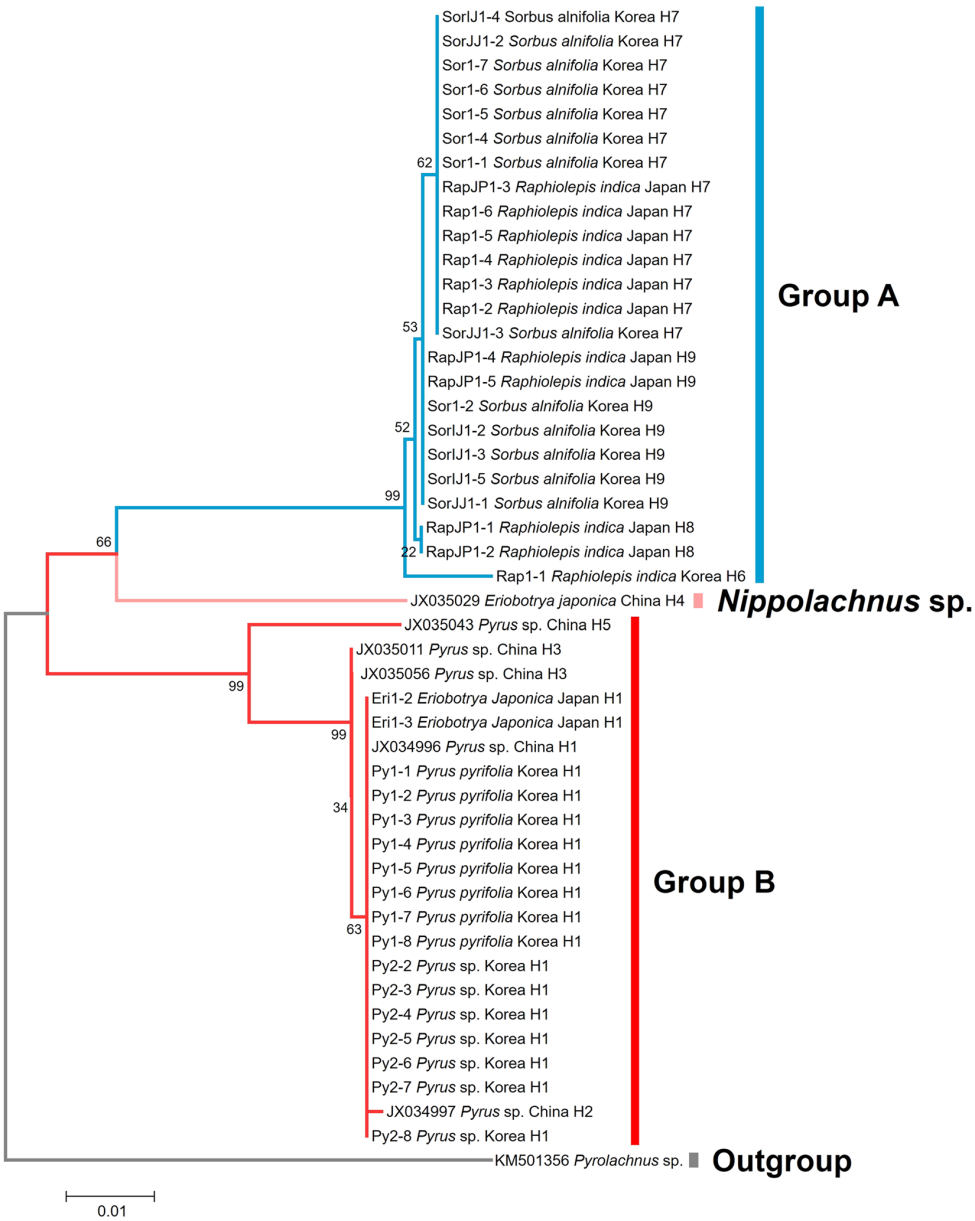
Results of the molecular analysis of the *Nippolachnus piri* complex.

A total of 41 new *COI* sequences of *Nippolachnus piri* from the four host-plant associated populations were produced in this study (S Table 1, S Fig. 1). All of the sequences are deposited in Genbank (MG333573–MG333613).

The overall mean genetic distance was 4.6% for the final dataset of 48 sequences of the *Nippolachnus piri* complex. Among the four different host-plant associated populations, the genetic distance ranged from 0% to 9.1%. The genetic distance between the *N*. *piri* populations on *Eriobotrya* and *Pyrus* (group B) and the *N*. *piri* populations on *Rhaphiolepis* and *Sorbus* (group A) ranged from 7.4% to 9.1%. *Nippolachnus* sp. (Genbank accession number: JX035029) and the group A and B showed 8.1–8.5% and 6.7–7.6% of genetic divergence, respectively. The genetic divergence in each group was 0–1.3% (group A) and 0–3.1% (group B), respectively. In the group B, one sequence (Genbank accession number: JX035043) was distinct from the other sequences with 2.8–3.1% of genetic divergence.

### Neighbour-joining tree

The neighbour-joining tree (NJ tree) that was inferred from the partial *COI* sequence showed that the *N*. *piri* complex was divided into two groups (Fig. 1). The two clades were *N*. *piri* populations on *Rhaphiolepis* and *Sorbus* (group A) and *N*. *piri* populations on *Eriobotrya* and *Pyrus* (group B). The two clades were clearly separated from each clade, which suggests that using the *COI* barcode region is effective for the identification of this species complex. The *COI* sequence of the group A was not related to the *COI* sequences of *N*. *piri* that were downloaded from Genbank (JX034996, JX034997, JX035011 and JX035056). *Nippolachnus* sp. on *Eriobotrya* (Genbank accession number: JX035029) formed a distinct clade that was separate from the two clades of *N*. *piri* (Fig. 1).

### Haplotype analysis

**Figure 2:**
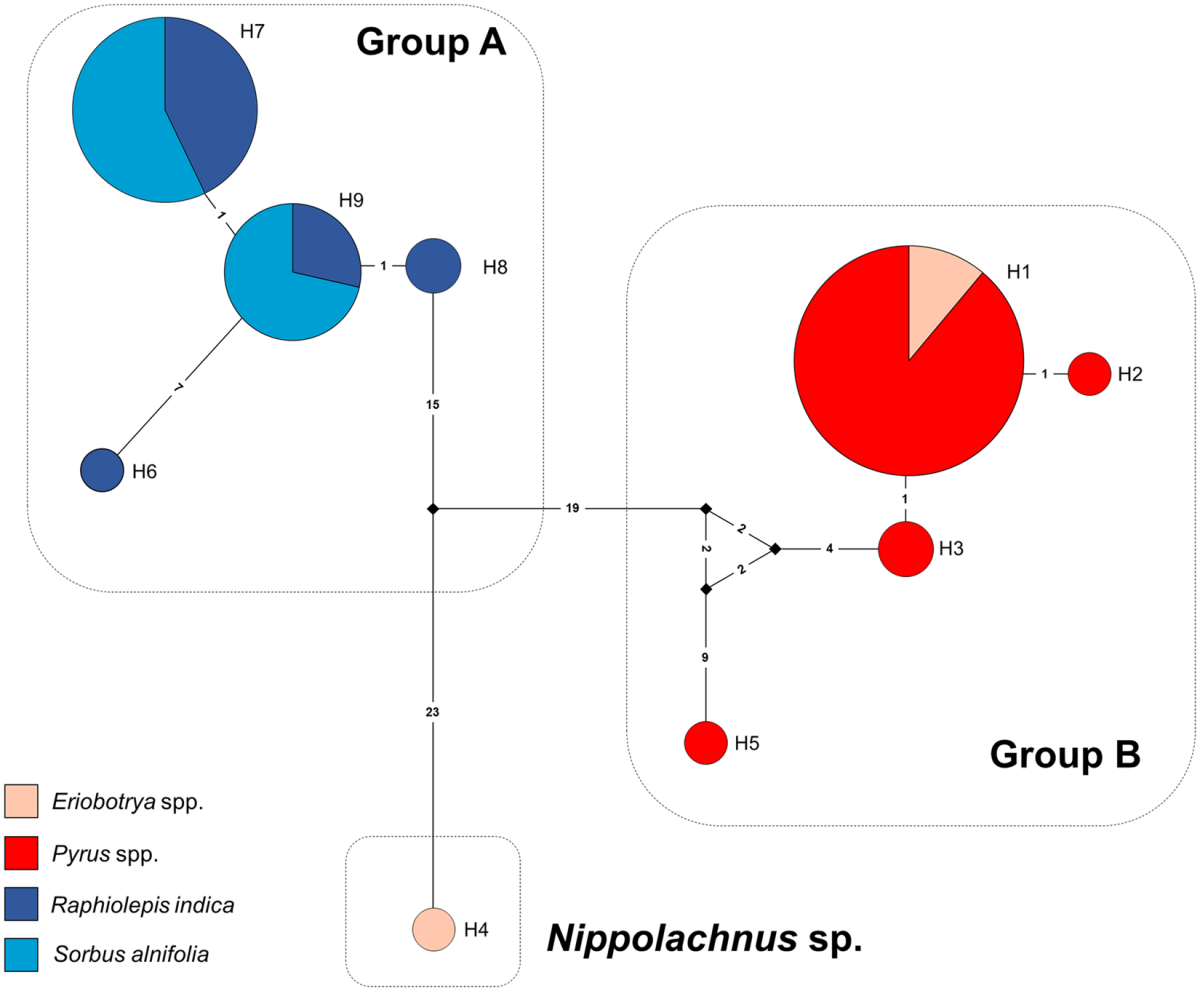
Haplotype network of the *Nippolachnus piri* complex.

From the 47 *COI* sequences of *Nippolachnus* spp., a total of nine haplotypes were identified (Fig. 2). Samples from the group A and B never included the same haplotype. The most frequently observed haplotype was H1 in the samples from every population of the group B (18 of 22 samples), whereas some haplotypes (H2, H3 and H5) were rarely observed. Samples on *Pyrus* were found to include four haplotypes (H1, H2, H3 and H5). Although haplotype H1 contained both the *N*. *piri* populations on *Eriobotrya* and *Pyrus*. *Nippolachnus* sp. on *Eriobotrya* (Genbank accession number: JX035029) was a unique haplotype (H4) on *Eriobotrya*. In the group A, H7 (14 of 24 samples) and H9 (7 of 24 samples) were observed most frequently. The samples on *Rhaphiolepis* were divided into four haplotypes (H6–H9). The *N*. *piri* populations on *Sorbus* were divided into two haplotypes (H7 and H9).

### Systematic accounts of the *Nippolachnus piri* species complex

Two of three species discussed here are supported by both molecular and morphological evidence and the differences between them are given in Table 1.

**Table 1:** Differences between apterous and alate viviparous females of *Nippolachnus piri* and *N*. *micromeli* stat. nov. “–” means that the feature does not apply to given morph.

Character	Apterous viviparous females		Alate viviparous females	
	*Nippolachnus piri*	*N*. *micromeli*	*Nippolachnus piri*	*N*. *micromeli*
Colour in life	Light green with green markings and brownish hind legs	Pale of whitish.	Brownish with brown legs and dark distal part of hind tibiae	Blackish with uniformly dark hind tibiae
Colour of mounted specimens	Antennae pale with brown distal part. Hind legs yellow or light brown with dark distal part of tibiae	Antennae uniformly pale. Hind legs pale with darker distal part of tibiae	Antennae brown to light brown. Hind femore yellow with darker distal part. Hind tibiae yellow or light brown.	Antennae dark brown. Hind femora light brown with dark half of length. Hind tibiae dark brown
HT I colour	dark	pale	dark	pale
Sclerotization of abdomen	—	—	ABD VI and VII without sclerites	ABD VI and VII with sclerites
Body length*	2.27–3.55	2.25–2.97	2.90–4.05	2.87–3.25
Antennae length	0.91–1.03 mm	0.75–0.84 mm	—	—
HT I b/HT I d	1.60–2.00	2.00–3.50	—	—
Hind legs length	3.88–4.74 mm	2.90–3.57 mm	—	—
SIPH pore diameter	0.12–0.13 mm	0.08–0.10 mm	—	—
PT/BASE	—	—	0.42–0.50	0.50–0.57
ARS/ANT III	—	—	0.48–0.51	0.43–0.46
ANT III rhinaria	—	—	8–11	5–7

*This character does not allow differentiation between taxa but helps to better recognize the selectivity of the antennae length character.

*Nippolachnus* Matsumura, 1917.

*Nippolachnus piri* Matsumura, 1917.

**Figure 3:**
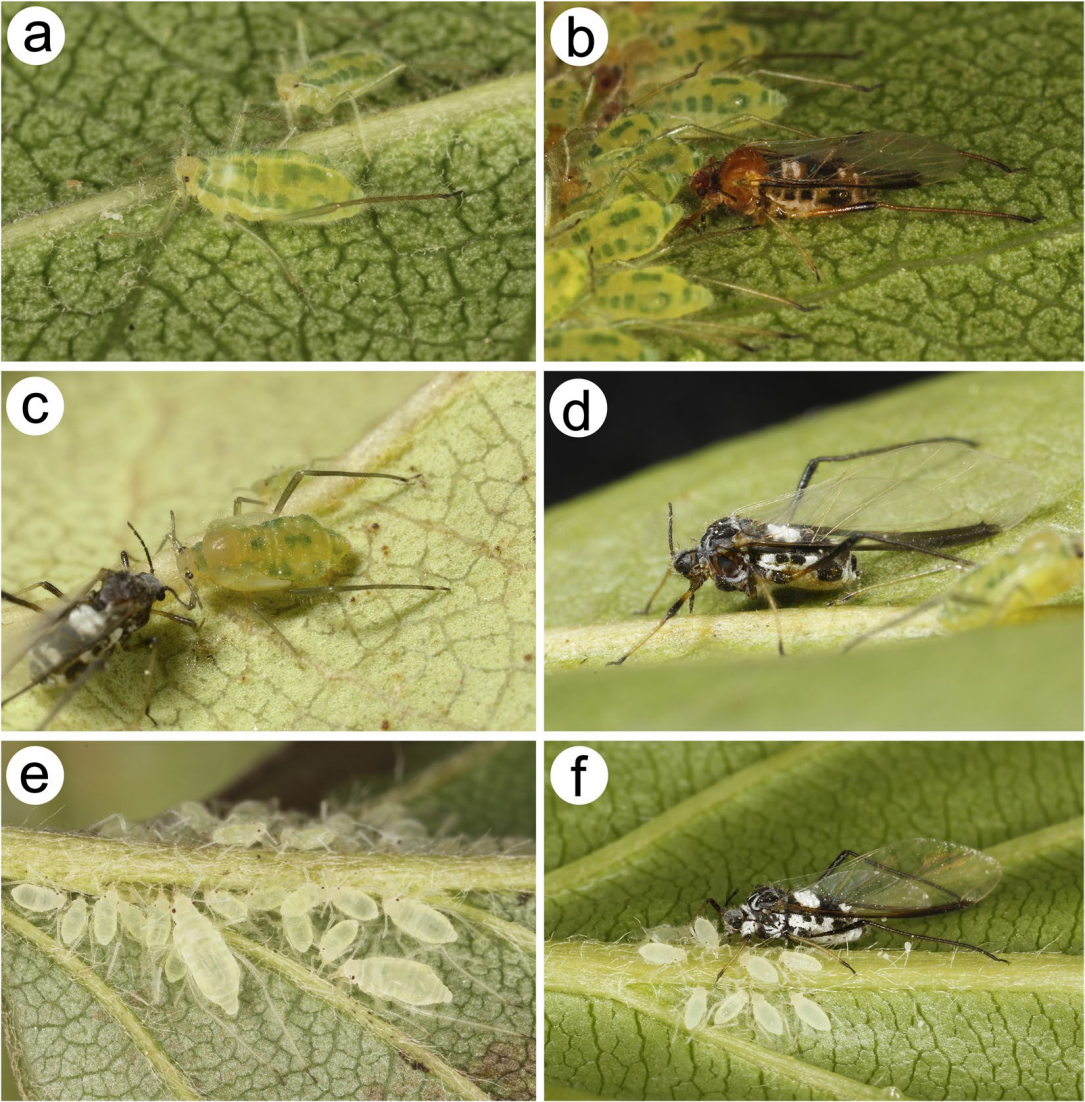
*Nippolachnus piri* complex representatives in life: (**a**) apterous viviparous females of *N*. *piri* on *Pyrus*: head yellow-light green, ANT pale to light green with light brown PT. Pronotum green, mesonotum and metanotum light green with green longitudinal marginal stripes, fore and middle legs pale green with light brown tarsi, hind legs with greenish femora, light brown tibiae and dark tarsi, abdomen light green with green longitudinal marginal stripes, spinal patches on ABD I, IV and V and green cross bars on ABD VII; (**b**) alate viviparous female of *N*. *piri* on *Pyrus* pigmentation: head brown, ANT dark. Pronotum dark brown, the rest of thorax brown, wings membranous, hyaline, fore and middle legs with brown femora, light brown tibiae and brown tarsi, hind legs with pale proximal part, brown central and dark brown distal part of femora, brown tibiae with dark distal part and dark tarsi, abdomen brown with dark sclerites, SIPH and white spino-pleural waxy stripes; (**c**) alate viviparous female and alatoid nymph of *N*. *micromeli* on *Rhaphiolepis*; (**d**) alate viviparous female of *N*. *micromeli* on *Rhaphiolepis*: head blackish to black (if covered by thin wax layer than dark grey). ANT black with lighter basal part of ANT III. Thorax black (if covered by wax then grey to white). Wings membranous, hyaline. Fore and middle legs with brown femora with yellowish proximal parts, light yellow tibiae with dark distal parts and brown tarsi. Hind legs with dark brown to black femora with sometimes yellow proximal part, dark brown to black tibiae tarsi. Abdomen dark with black sclerites and white waxy spino-pleural stripes; (**e**) apterous viviparous females and nymphs of *N*. *micromeli* on *Sorbus*: whitish to creamy-yellowish. SIPH very pale. Sometimes very pale greenish, small patch can be noted on abdomen. Legs as pale as the rest of body with only distal part of hind tibiae and tarsi brown; (**f**) alate viviparous female and nymphs of *N*. *micromeli* on *Sorbus*.

**Figure 4:**
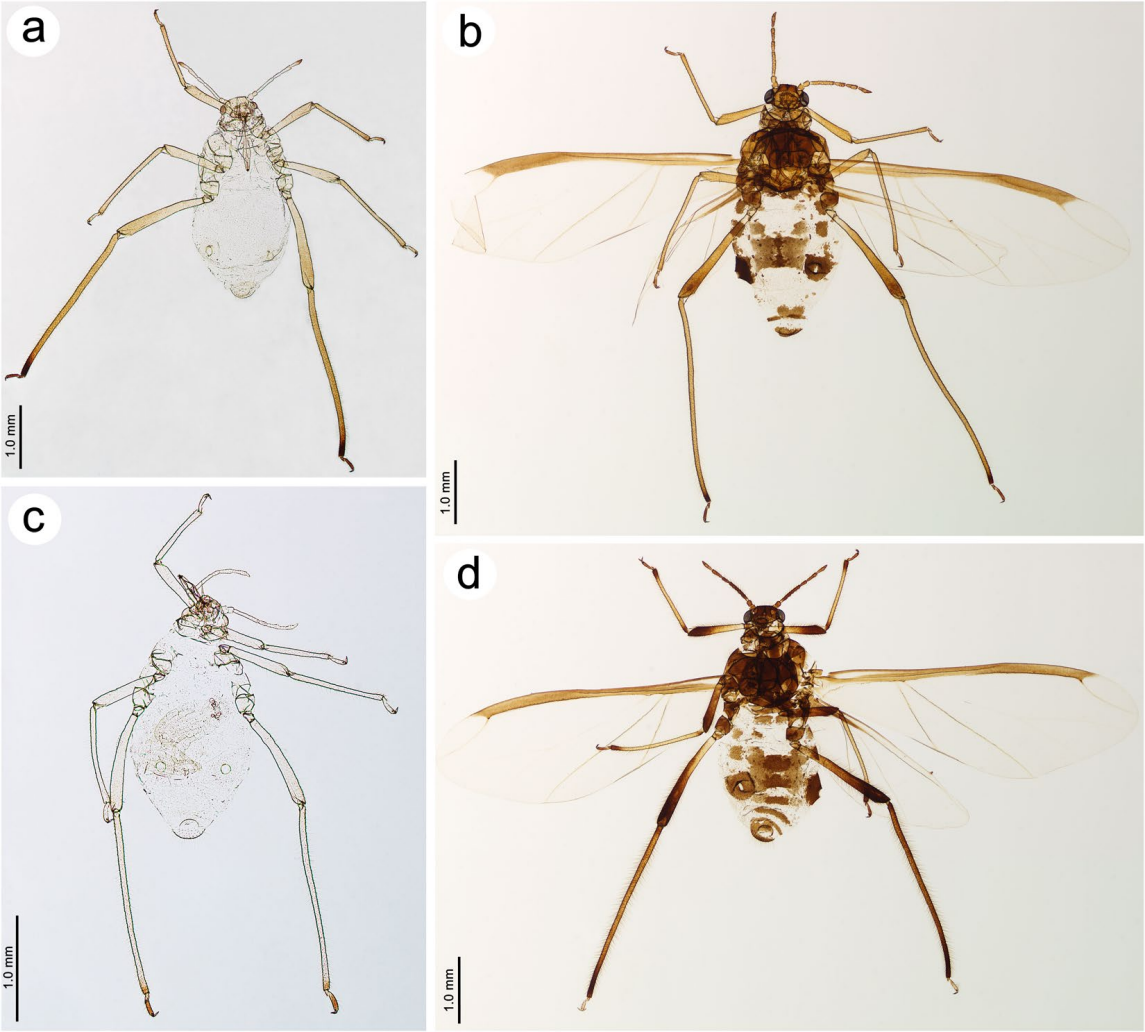
*Nippolachnus piri* complex members: (**a**) apterous viviparous female of *N*. *piri*; (**b**) alate viviparous female of *N*. *piri*; (**c**) apterous viviparous female of *N*. *micromeli*, (**d**) alate viviparous female of *N*. *micromeli*.

**Apterous viviparous female** – redescription.

(Figs 3 and 4; S Figs 2 and 3; S Table 2).

#### Colour

Life specimens green (Fig. 3a), mounted specimens: body in general pale with yellow head, pale yellow ANT with light brown apex of ANT V, BASE and PT. Fore and middle legs uniformly pale yellow to yellow (Fig. 4a), hind legs with yellow femora and yellow to light brown tibia with dark distal parts and whole tarsi (S Fig. 2a,c; 3a,c). ***Morphological characters*:** HW 0.54–0.62 × ANT. Head densely covered by long, very fine and pointed setae. ANT 0.26–0.42 × BL. ANT III with 0–1 secondary rhinarium, ANT IV always shorter than ANT V. ANT V always shorter than ANT VI. ANT VI PT 0.41–0.54 × BASE with five accessory rhinaria. Other antennal ratios: VI:III 0.52–0.60, V:III 0.47–0.56, IV:III 0.30–0.41. ANT covered by numerous long, very fine and pointed setae. LS III 4.01–7.50 × BD III. URS 0.56–0.60 × ANT III, 0.97–1.11 × ANT VI and 0.70–0.86 × HT II with eight accessory setae. Hind legs covered by long, fine and pointed setae, 0.10–0.18 mm long. First segments of tarsi all with 1-1-1 “sense pegs” and 10–12 ventral setae. HT II 0.67–0.80 × ANT III and 1.14–1.47 ANT VI. SIPH on low, poorly sclerotised and setose sclerites. Dorsal setae long, very fine and pointed, 0.11–0.15 mm long.

**Figure 5:**
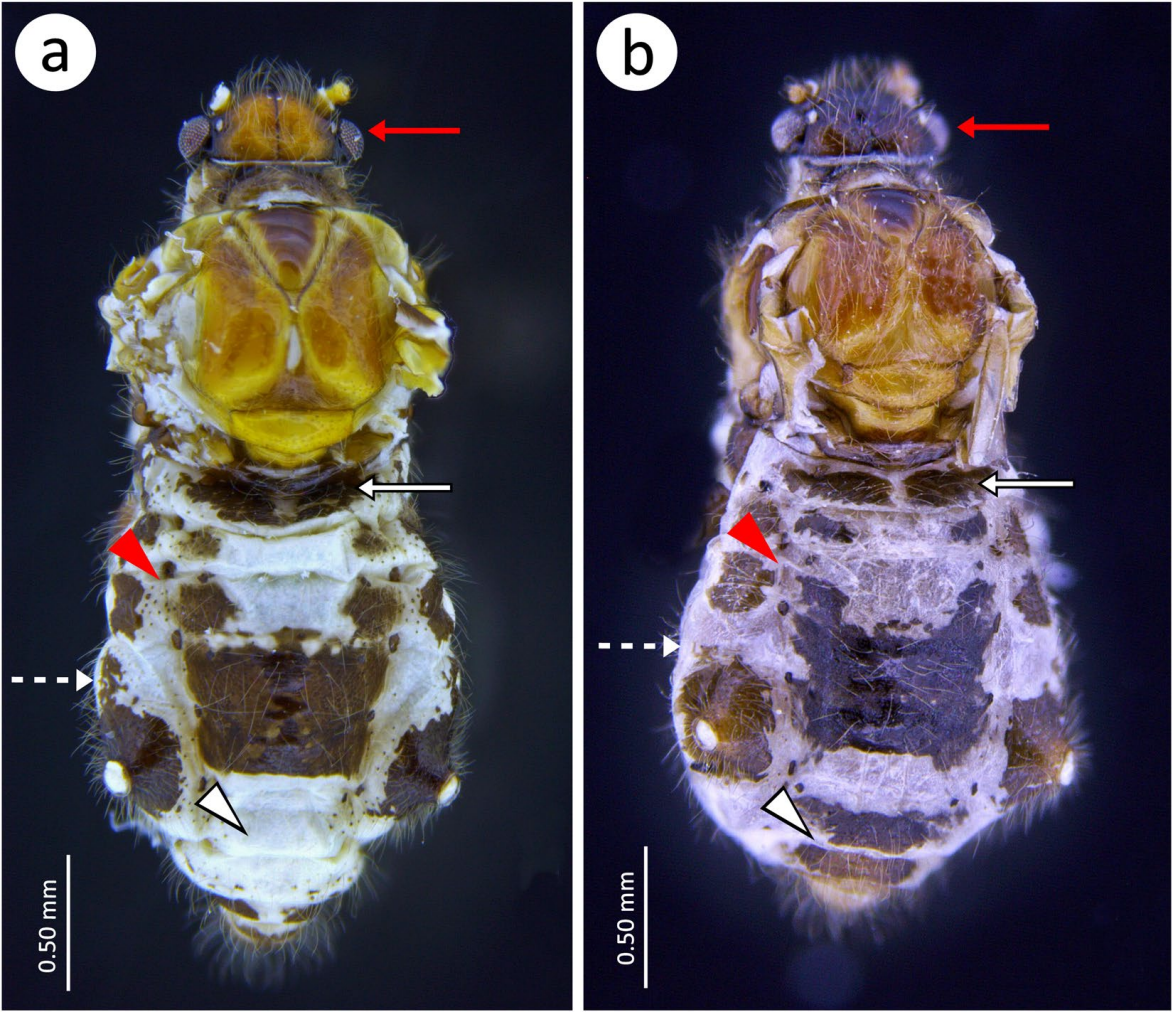
Comparison of alate viviparous females sclerotisation of *Nippolachnus piri* complex: (**a**) *N*. *piri*; (**b**) *N*. *micromeli*; red arrows – differences in the head pigmentation, white arrow – differences in A the BD I cross-bar, red arrowhead – differences in the sclerotisation on ABD II and ABD III, white dotted arrow – differences in the sclerotisation on ABD IV, white arrowhead – presence of cross-bars on ABD VI and ABD VII.

**Alate viviparous female**– redescription.

(Figs 3–5; S Fig. 4; S Table 2).

#### Colour

Life specimens brownish (Fig. 3b), mounted specimens: body with brown head, yellowish-brown ANT with brown apex of ANT IV, ANT V and VI (S Fig. 4a). Fore and middle legs uniformly pale yellow to yellow, hind legs with yellow femora and yellow to light brown tibia with dark distal parts and whole tarsi (Fig. 4b). ***Morphometric characters***: HW 0.59–0.65 × ANT. Head densely covered by long, very fine and pointed setae. ANT 0.28–0.33 × BL. ANT III with 8–11 big rounded or oval and rather transverse secondary rhinaria, ANT IV shorter than ANT V with 2–3 big secondary rhinaria. ANT V shorter than ANT VI with 0–1 rounded secondary rhinarium. ANT VI with PT 0.42–0.50 × BASE, 0–1 small rounded secondary rhinarium and 5–6 accessory rhinaria (S Fig. 4a). Other antennal ratios: VI:III 0.55–0.60, V:III 0.47–0.55, IV:III 0.35–0.38. ANT covered by numerous long, very fine and pointed setae. LS III 3.20–5.20 × BD III. URS 0.48–0.57 × ANT III, 0.80–1.00 × ANT VI and 0.70–0.90 × HT II with eight accessory setae. Fore wings membranous, media one branched. The ramification of media starts together with the RS branch point. Minute scales distribution on the wing membrane only in the distal part of the wing (S Fig. 4d). Hind legs covered by long, fine and pointed setae, 0.13–0.21 mm long. First segments of tarsi all with 1-1-1 “sense pegs” and 12 ventral setae. HT II 0.61–0.77 × ANT III and 1.06–1.33 ANT VI. SIPH on low, well developed, sclerotised and setose sclerites. Dorsal setae long, very fine and pointed, 0.11–0.14 mm long. Abdominal sclerotisation: ABD I with spino-pleural cross band and small marginal sclerites, ABD II and III with pleural and marginal sclerites and very small sclerites at setal bases between them, ABD IV with marginal sclerites more or less fused with SIPH sclerites and big sclerotic spino-pleural cross band, ABD V proximal part with spino-pleural cross band fused with the previous one, ABD VI and VII without sclerites, ABD VIII with pleuro-marginal cross band (Fig. 5a). Less sclerotised part of the patch on ABD IV and V cuticle wrinkled or rugose in form of polygonal reticulation (S Fig. 4c).

*Nippolachnus micromeli* Shinji, 1924 **stat**. nov.

*Nippolachnus piri* Tao, 1962^18^, Eastop and Hille Ris Lambers, 1976^19^; Remaudière and Remaudière, 1997^37^.

**Apterous viviparous female** – redescription.

(Figs 3 and 4; S Figs 2 and 3; S Table 2).

#### Colour

Life specimens: body very pale (Fig. 3e),mounted specimens: body pale, almost colourless, only the legs are very pale yellowish with the same pale ANT. III TIBIAE pale yellowish with distal part light brown, pale HT I and the very proximal part of HT II and brown remainder HT II (Fig. 4c, S Fig. 2b,d; 3b,d). ***Morphological characters***: HW 0.55–0.64 × ANT. Head densely covered by long, very fine and pointed setae. ANT 0.26–0.37 × BL. ANT IV shorter than ANT V. ANT V shorter than ANT VI. ANT VI PT 0.45–0.64 × BASE with 5–6 accessory rhinaria. Other antennal ratios: VI:III 0.53–0.66, V:III 0.51–0.58, IV:III 0.25–0.34. ANT covered by numerous long, very fine and pointed setae. LS III 4.33–6.00 × BD III. URS 0.48–0.60 × ANT III, 0.80–1.07 × ANT VI and 0.63–0.78 × HT II with eight accessory setae. Hind legs covered by long, fine and pointed setae, 0.07–0.16 mm long. First segments of tarsi all with 1-1-1 “sense pegs” and 10–12 ventral setae. HT II 0.70–0.84 × ANT III and 1.22–1.57 ANT VI. SIPH on low, poorly sclerotised and setose sclerites. Dorsal setae long, very fine and pointed, 0.11–0.14 mm long.

**Alate viviparous female** – redescription.

(Figs 3–5; S Fig. 5; S Table 2).

#### Colour

Life specimens dark brown to blackish (Fig. 3c,d,f),mounted specimens: body with dark brown head, brown ANT with only slightly lighter basal part of ANT III (S Fig. 5a). Fore and middle legs with yellow femora with dark brown distal parts, yellow tibiae with brown proximal and distal ends and tarsi. Hind legs with dark brown femora with yellow proximal parts, yellow to light brown tibia with dark distal parts and light brown tarsi (Fig. 4d). ***Morphometric characters***:HW 0.56–0.61 × ANT. Head densely covered by long, very fine and pointed setae. ANT 0.31–0.34 × BL. ANT III with 5–7 (seven only very rarely and on one segment) big and rather rounded secondary rhinaria. ANT IV shorter than ANT V with 2 big secondary rhinaria. ANT V slightly longer or shorter than ANT VI with 0–2 rounded secondary rhinaria. ANT VI with PT 0.50–0.57 × BASE with 5–6 accessory rhinaria on PT (S Fig. 5b). Other antennal ratios: VI:III 0.51–0.64, V:III 0.50–0.62, IV:III 0.34–0.40. ANT covered by numerous long, very fine and pointed setae. LS III 4.75–6.00 × BD III. URS 0.43–0.46 × ANT III, 0.73–0.85 × ANT VI and 0.65–0.73 × HT II with eight accessory setae. Fore wings membranous, media one branched. The ramification of media starts together with the start of pterostigma. Minute scales distribution on the wing membrane starts from the middle part of the wing (S Fig. 5d). Hind legs covered by long, fine and pointed setae, 0.08–0.17 mm long. First segments of tarsi all with 1-1-1 “sense pegs” and 12 ventral setae. HT II 0.60–0.71 × ANT III and 1.07–1.23 ANT VI. SIPH on low, well developed, sclerotised and setose sclerites. Dorsal setae long, very fine and pointed, 0.11–0.14 mm long. Abdominal sclerotisation: ABD I with two spino-pleural sclerites, without marginal sclerites, ABD II and III with pleural and marginal sclerites, without sclerites at setal bases between them, ABD IV without marginal sclerites and with big sclerotic spino-pleural cross band, ABD V proximal part with spino-pleural cross band fused with the previous one, ABD VI and VII with spinal sclerites, ABD VIII with pleuro-marginal cross band (Fig. 5b). Less sclerotised part of the patch on ABD IV and V cuticle wrinkled or rugose without polygonal reticulation (S Fig. 5d).

### Key to known apterous viviparous females of the genus *Nippolachnus*

(apterous viviparous females of *N*. *xitianmushanus* undescribed).

**1**. Legs dark brown to black … ***N***. ***himalayensis***

- Legs pale to yellow, except that sometimes hind tibiae are light brown with brown apices and tarsi … **2**.

**2**. Distal part of hind tibiae and tarsi pale or yellow. The length from basal part of ANT VI to accessory rhinaria as long as or shorter than the length to the major rhinarium … ***N***. ***bengalensis***.

- Distal part of hind tibiae and tarsi brown to dark. The length from basal part of ANT VI to accessory rhinaria as long as or longer than the length to the major rhinarium … **3**.

**3**. HT I and whole HT II brown to dark. ANT VI with light brown PT. HT I basal length/HT I dorsal length 1.60–2.00… ***N***. ***piri***.

- HT I and basal part of HT II pale to yellow. ANT VI with pale PT. HT I basal length/HT I dorsal length 2.00–3.50 … ***N***. ***micromeli*** stat. nov.

**Key to alate viviparous females of the genus** ***Nippolachnus***.

**1**. Media of forewing twice branched. ANT III with numerous small to medium-sized rhinaria … ***N***. ***himalayensis***.

- Media of forewing once branched. ANT III with no more than 15 medium-sized to large secondary rhinaria … **2**.

**2**. Secondary rhinaria on ANT VI on BASE, at the most one secondary rhinarium on PT. Sculpture of the dorsal abdominal patch crumpled or wrinkled irregularly, not forming polygonal arrangement … ***N***. ***bengalensis***.

**-** Secondary rhinaria all on PT, at most one secondary rhinarium on BASE. Sculpture of the dorsal abdominal patch smooth or wrinkled and forming polygonal arrangement … **3**.

**3**. Marginal sclerotization on ABD VII in form of visible marginal sclerites … ***N. xitianmushanus***.

**-** Marginal sclerotization completely absent or only in form of separate small sclerites at setal bases … **4**.

**4**. ABD VI and VII without spino-pleural cross bars, ANT III wit 8–11 secondary rhinaria, ARS/ANT III 0.46–0.58 … ***N***. ***piri***.

**-** ABD VI and VII with spino-pleural cross bars, ANT III with 5–7 secondary rhinaria, ARS/ANT III 0.43–0.46 … ***N***. ***micromeli*** stat. nov.

### Morphology and sensilla of the *Nippolachnus piri* complex

#### General morphology

**Figure 6:**
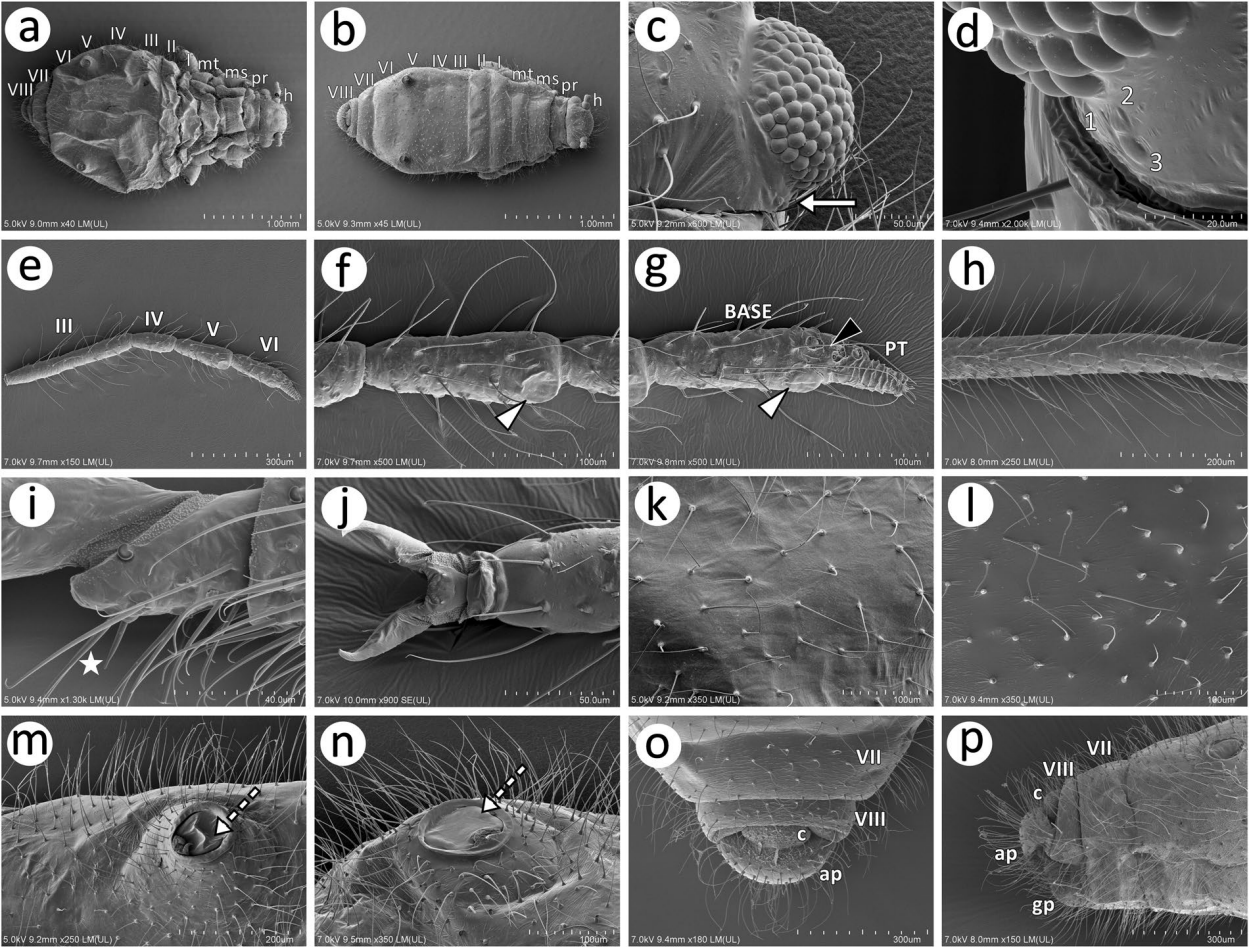
SEM of general characters of the morphology of *Nippolachnus piri* complex: (**a**) apterous viviparous female of *N*. *piri*; (**b**) apterous viviparous female of *N*. *micromeli*; (**c**) lateral side of the head of an alate viviparous female of *N*. *piri* with a large compound eye and a small triommatidium under the eye (white arrow); (**d**) residual triommatidium under the compound eye in an apterous viviparous female of *N*. *micromeli*; (**e**) antennal flagellum of *N*. *piri* with long and fine setae; (**f**) ANT V of *N*. *piri* with a primary rhinarium (white arrowhead); (**g**) ANT VI of *N*. *piri* with primary rhinaria: major rhinarium (white arrowhead) and accessory rhinaria (black arrowhead); (**h**) hind tibia chaetotaxy of *N*. *micromeli*; (**i**) first segment of the hind tarsus of *N*. *micromeli* with one sense peg (star); (**j**) end of the second segment of the hind tarsus of *N*. *micromeli* with normal-shaped and pointed claws; (**k**) dorsal side of the abdomen and chaetotaxy of an apterous viviparous female of *N*. *piri*; (**l**) dorsal side of the abdomen and chaetotaxy of an apterous viviparous female of *N*. *micromeli*; (**m**) SIPH of *N*. *piri* with well visible operculum (dotted arrow); (**n**) SIPH of *N*. *micromeli* with well visible operculum (dotted arrow); (**o**) dorsal end of the abdomen of *N*. *micromeli*; (**p**) lateral end of the abdomen of *N*. *micromeli*.

Representatives of the *Nippolachnus piri* complex are pear-shaped or elongate oval aphids with a setose body and appendages. The head is separate from the pronotum. All of the tergites of the thorax and ABD I and II are separated. ABD III–VI are fused. Weakly developed sutures between ABD tergites are visible in *N*. *micromeli* or else the area of abdomen is completely smooth in *N*. *piri*. ABD VII and VIII are also separated (Fig. 6a,b). Apterous and alate viviparous females are characterised by a head with normal compound eyes with many facets being rather tightly adjoined, 0.13–0.17 μm in diameter. On the lateral side of the head under the compound eye in both morphs, a small more or less developed triommatidium is present (Fig. 6c,d). The antennae are six-segmented. The ANT flagellum comprises four segments with long, fine and pointed setae (type I trichoid sensilla) (Fig. 6e). On the apex of ANT V and on the end of the BASE of ANT VI, a medium-sized rounded primary rhinarium is present (Fig. 6f). Five or six small rounded accessory rhinaria were also observed on the end of the BASE and PT of ANT VI. ANT VI bears two kinds of setae: long, fine and pointed on the BASE and very short, rigid and slightly pointed on the apex of PT (Fig. 6g). The legs are densely covered by long and very long fine and pointed setae (Fig. 6h). HT I is rather short with a very short dorsal side without setae. It has one “sense peg” and a few long fine and pointed setae on the ventral side (Fig. 6i). HT II is characterised by normal-shaped claws and extremely short parempodia (empodial setae) in the form of slightly developed pegs (Fig. 6j). The dorsal side of the body is covered by long and very long fine and pointed setae. The dorsal cuticle is smooth in *N*. *piri* (Fig. 6k) and more or less wrinkled in *N*. *micromeli* (Fig. 6l). Siphunculi lie on low rounded and setose sclerites on ABD V. The siphuncular aperture is surrounded by a well-developed, rolled-up flange and are covered on the entire diameter by an operculum (Fig. 6m,n). The perianal area is densely covered by long, fine but rigid and pointed setae. The cauda is small and rounded, the anal plate is rounded, especially from the dorsal side (Fig. 6o), and is slightly shifted up (Fig. 6p). In alate viviparous females, the wings are characterised by once branched media. The wing membrane is less covered by small minute scales in *N*. *piri* and on almost the entire area in *N*. *micromeli* (S Fig. 6).

#### *Antennal* sensilla

**Figure 7:**
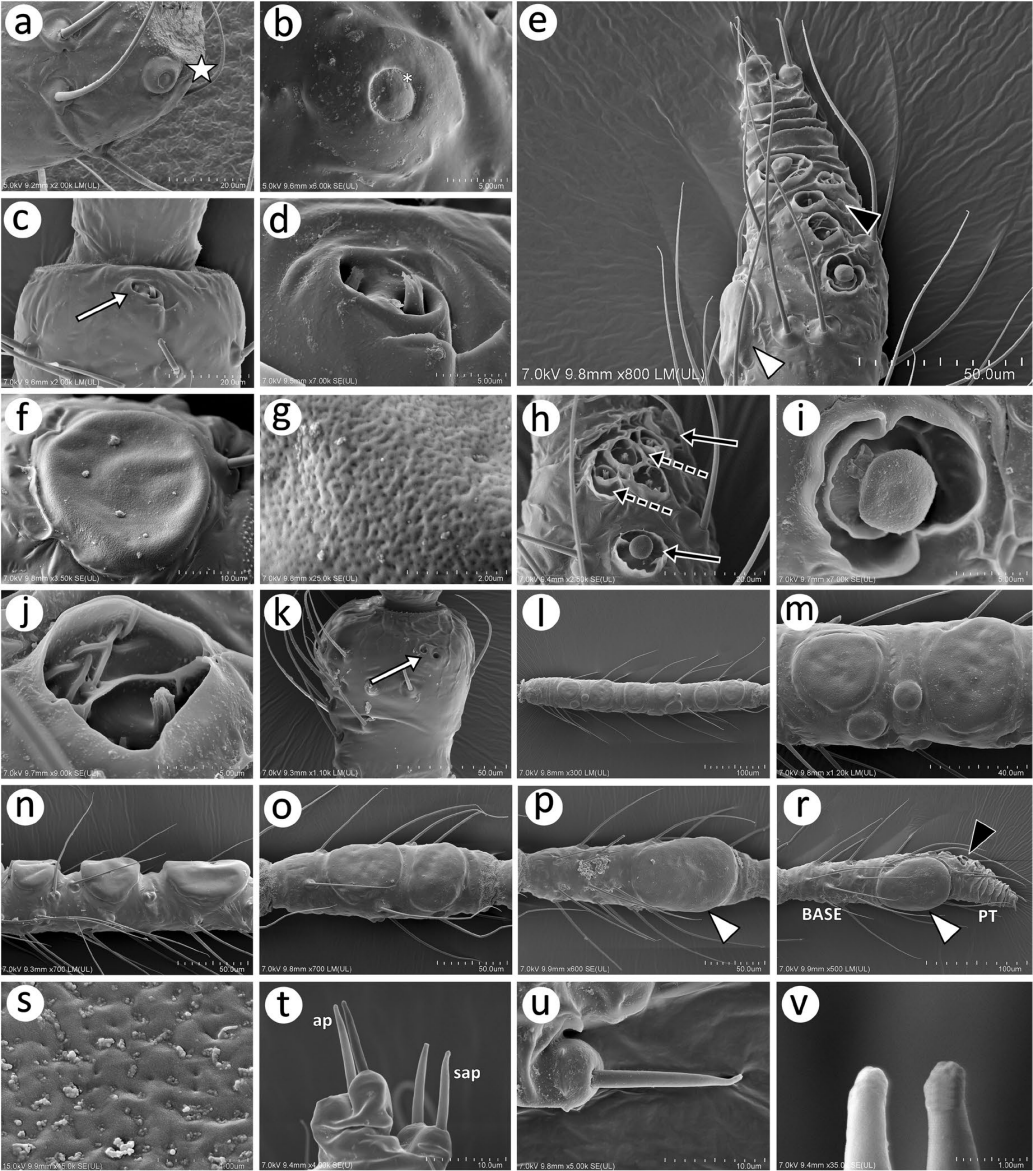
SEM of antennal sensilla in *N*. *piri* complex: (**a**) pedicel of apt. of *N*. *piri* with campaniform sensillum (star); (**b**) campaniform sensillum on the pedicel of al. of *N*. *piri*; (**c**) pedicel of apt. of *N*. *micromeli* with two rhinariola; (**d**) fine structure of rhinariola of *N*. *micromelli* showing slightly tuberculate base and two kinds of pegs; (**e**) primary rhinaria on ANT VI of *N*. *piri* with a rounded major rhinarium (large placoid sensillum) (white arrowhead) and accessory rhinaria (small placoid and coeloconic sensilla) (black arrowhead); (**f**) fine structure of a large placoid sensillum on ANT V of *N*. *micromeli*; (**g**) porous surface of a placoid sensillum of *N*. *micromeli*; (**h**) arrangement of the accessory rhinaria on the PT of *N*. *micromeli* coeloconic sensilla (dotted arrows) between small placoid sensilla (solid arrow); (**i**) fine structure of the small placoid sensillum of *N*. *piri*; (**j**) fine structure of the coeloconic sensillum of *N*. *piri* with long projections; (**k**) pedicel of al. of *N*. *micromeli* with two separate rhinariola; (**l**) ANT of al. of *N*. *piri* with long and fine trichoid sensilla and placoid sensilla (secondary rhinaria); (**m**) fine structure of the placoid sensilla (secondary rhinaria) on ANT III of al. of *N*. *piri*; (**n**) lateral side of the protuberant placoid sensilla (secondary rhinaria) of al. of *N*. *micromeli*; (**o**) placoid sensilla (secondary rhinaria) on ANT IV of al. of *N*. *piri*; (**p**) large placoid sensillum (primary rhinarium) (white arrowhead) on ANT V of al. of *N*. *piri*; (**r**) large placoid sensillum (primary rhinarium) (white arrowhead) and accessory rhinaria (black arrowhead) on ANT VI of al. of *N*. *piri*; (**s**) multiporous surface of the large placoid sensillum of ANT VI of al. of *N*. *piri*; (**t**) trichoid sensilla on the apex of PT of *N*. *micromeli* arranged into the apical setae (ap) and subapical setae (sap); (**u**) fine structure of the trichoid sensillum on the apex of PT of *N*. *piri*; (**v**) apices of the trochoid sensilla on the apex of PT of *N*. *micromeli*.

The antennal sensilla of the apterous and alate viviparous females of the *Nippolachnus piri* complex are distributed on all segments and are divided into: trichoid sensilla (type I and type II trichoid sensilla), multiporous placoid sensilla (big placoid sensilla and small placoid sensilla), coeloconic sensilla, rhinariola and campaniform sensilla. Type I trichoid sensilla are numerous and are present on all the ANT segments (ANT I-ANT VI BASE). The pedicel bears two types of sensilla. On the dorso-lateral side of apex one rounded campaniform sensillum is visible. This sensillum is characterised by quite wide external ring and a small internal one with very small pore (Fig. 7a,b). Also, near the apex on the ventral side of this segment, there is well visible double rhinariolum (Fig. 7c). The rhinariolum is oval, 9–10 μm long and 4–5 μm wide with well-developed, slightly raised edges and two columnar pegs with four or five projections, which are separated by a wide partition (Fig. 7d). Big multiporous placoid sensilla are present on the apex of ANT V and the BASE (Fig. 7e,f). In the apterous morphs, they are rounded, slightly protuberant with gentle edges and 35–40 pores per 1 μm^2^ (Fig. 7g). On the upper boundary of the BASE and on the PT, there are two kinds of sensilla: small multiporous placoid sensilla and sunken coeloconic sensilla (Fig. 7f,h). The small placoid sensilla are located on the polar positions of the group of sensilla (Fig. 7e), are mushroom-shaped and lie in small cavities, surrounded by well-developed sclerotic flanges (sometimes double) (Fig. 7i). The sunken coeloconic sensilla are always located between the placoid sensilla and are developed in the form of three or four columnar pegs with 12–14 projections and are surrounded by very well-developed flanges. The pedicel of alate viviparous female also bears double rhinariolum (Fig. 7k). Antennae of alatae females are further characterised by the presence of multiporous placoid sensilla (secondary rhinaria) that are always on ANT III and IV and sometimes also on ANT V. The multiporous placoid sensilla in the *Nippolachnus piri* complex are distributed along the entire length of ANT III and IV, mostly in one row. The sensilla are large, rounded and nearly as wide as the width of the segment. Moreover, a few much smaller rounded multiporous sensilla could be observed between the larger ones (Fig. 7l,m,o). The placoid sensilla in *N*. *piri* are very flat whereas in *N*. *micromeli*, they are clearly protuberant (Fig. 7n). The placoid sensilla on ANT V and VI are very large, oval and wider than the width of segment (Fig. 7p,r). They also appear to be less porous (12–15 pores per 1 μm^2^) (Fig. 7s). In addition to the type I trichoid sensilla on the apical part of PT, five type II trichoid sensilla are present, which are divided into two groups – three and two slightly lower (Fig. 7t). They are very short, rigid, arise from rounded and protuberant sockets (Fig. 7u) and have rounded apices (Fig. 7v).

#### Other sensilla

**Figure 8:**
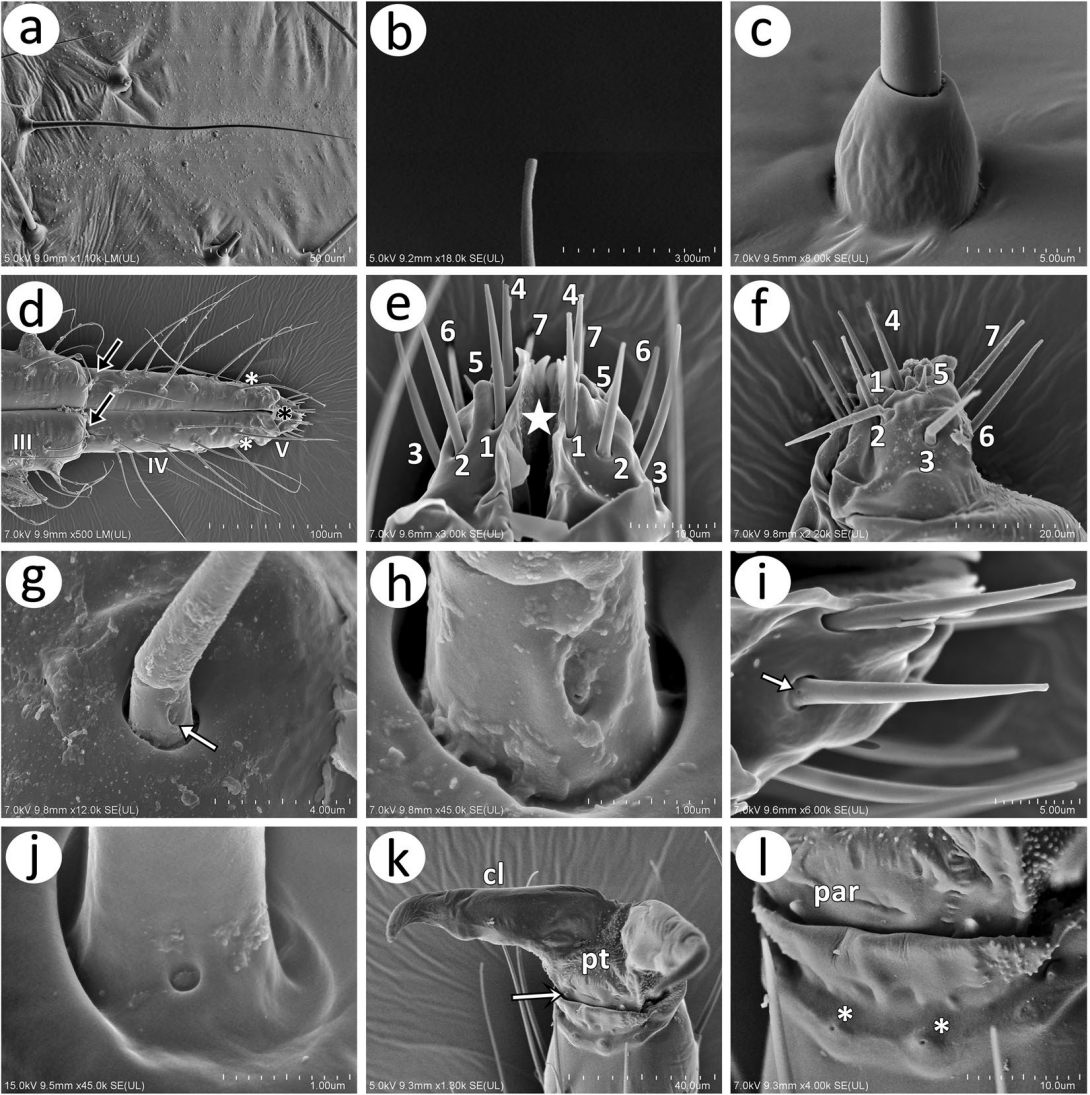
Body sensilla in *N*. *piri* complex: (**a**) fine structure of the trichoid sensillum covering body and legs of *N*. *piri*; (**b**) blunt apex of the trichoid sensillum of *N*. *piri*; (**c**) socket of trichoid sensillum of *N*. *micromeli*; (**d**) apical rostral segments of the mouthparts of *N*. *micromeli* covered by numerous long and fine type I trichoid sensilla on segments III and IV, type II basiconic sensilla on segment IV (black arrows), three pairs of type I trichoid sensilla forming the primary setae (white asterisk) and type III basiconic sensilla on segm. V (black asterisk); (**e**) ventral side of URS segment V of *N*. *piri* with seven pairs of type III basiconic sensilla; (**f**) lateral side of URS segment V of *N*. *piri* with type III basiconic sensilla showing that one pair is much shorter than the remaining sensilla; (**g**,**h**) fine structure of the basal part of the basiconic sensillum on URS V of *N*. *piri* with very small pores inside an oval cavity (arrow); (**i**) fine structure of basiconic sensillum on URS V of *N*. *micromeli* with rounded pores (arrow); (**j**) shape of the pores on the basal part of the basiconic sensillum on URS V of *N*. *micromeli*; (**k**) distal part of HT II of *N*. *piri* with normal-shaped, pointed claws (cl) and a pretarsus (pt) with extremely small parempodia (arrow); (**l**) residual parempodia (par) on the pretarsus of *N*. *micromeli* and semispherical structures with pores (asterisk).

The body of the *Nippolachnus piri* complex species is densely covered by long, very fine and in general pointed setae (type I trichoid sensilla), which at a higher magnification were observed to have blunt apices (Fig. 8a,b). The trichoid sensilla on the body all lie in high, semi-oval sockets (Fig. 8c). The apical rostrum segments are also densely covered by long, fine and pointed setae. Segment III is only covered by type I trichoid sensilla. Segment IV has one pair of short and pointed type II basiconic sensilla, about eight pairs of type I trichoid sensilla (accessory setae) and three pairs of type I trichoid sensilla on its distal part (primary setae) (Fig. 8d). The very short segment V is covered by seven pairs of very short, rigid and slightly pointed or blunt type III basiconic sensilla (Fig. 8e) of which the fifth pair is much shorter than the others (Fig. 8f). The basiconic sensilla lie in rather wide, rounded sockets (Fig. 8g). A clear cavity is visible on the basal part of each sensillum. In *N*. *piri*, the cavity is oval with very small pores (Fig. 8g,h), whereas in *N*. *micromeli* the basal parts of those sensilla are characterised by a rounded aperture (Fig. 8i,j). The pretarsus is characterised by extremely short, almost residual parempodia and a ring with sensilla-like structures with pores (Fig. 8k,l).

## Discussion

To date, *Nippolachnus* is one of the most recognizable genera of Asiatic Lachninae. Representatives of this genus are very characteristic due to the features of the apterous viviparous females, which are almost always pale coloured except for *N*. *himalayensis*, which is dirty greyish to brick red^14^. Moreover, the feeding site (the undersides of leaves) of both the parthenogenetic and sexual generations is unique and is unlike other deciduous-feeding lachnids (Lachnini, Stomaphidini and the remaining Tuberolachnini), which are known to feed on woody or sometimes the green parts (bark of trunks, branches and green stems) of various trees and shrubs^12^. There was also another important character of this genus, which was treated for some time as a tribal level feature of the former Nippolachnini^38^ – the apparent absence of triommatidia (unique character of aphids). This unusual feature has been emphasised for years by many authors when characterising *Nippolachnus*^11,12,20,39,40^. A detailed morphological study of the type species *N*. *piri* as well as of the cryptic species showed that triommatidia are present on the head but not on the ocular tubercle like in remaining aphids but is situated under the compound eye (Fig. 6c,d). This confirms that *Nippolachnus* is a member of Tuberolachnini despite its morphological distinctiveness^3^.

The results of molecular analyses corresponded with the morphological comparison. The four host-plant associated *N*. *piri* populations were clearly divided into the two groups (populations on *Rhaphiolepis* + *Sorbus* and populations on *Eriobotrya* + *Pyrus*) with 7.4% to 9.1% of a high inter-group genetic divergence. The two genetically distinct groups were only separated by their host-plant association not by geographical regions. This result suggests that *N*. *piri* on *Rhaphiolepis* and *Sorbus* (group A) is a cryptic species that has a different host-plant association. The cryptic species of *N*. *piri* may have diverged by a change in their host-plant association. A different host-plant association has been known to be a critical speciation factor for various herbivores including aphids^5^.

Until now, the *N*. *piri* complex has been recorded from Southeast (India, Laos, Taiwan, Vietnam and partial regions of China and Japan) to East Asia (China, Japan and Korea)^12,23^. In Southeast Asia, *N*. *piri* is usually observed on *Eriobotrya* spp.^41,42^ and *Pyrus* spp^11,23,43^, whereas in East Asia, this species is also commonly collected on *Rhaphiolepis* spp^22^ and *Sorbus alnifolia*^44^. Thus, we assume that the cryptic species may only be distributed within East Asia, although such an assumption needs to be interpreted with caution due to the restricted sampling.

Interestingly, the populations on *Eriobotrya* and *Pyrus* (H1) and the populations on *Rhaphiolepis* and *Sorbus* (H7 and H9) had the same haplotype within each group. This result may indicate that the host-plant range of *N*. *piri* (between *Eriobotrya* and *Pyrus*) and ***N. micromeli*** (between *Rhaphiolepis* and *Sorbus*) might have broadened more recently. Based on the morphological and molecular results, the two-species group appears to be an oligophagy. However, additional ecological studies are needed to clarify whether the two species are oligophagous species. In *N*. *bengalensis*, the host-plant association is similar to *N*. *piri*. In addition to *Photinia*, *N*. *bengalensis* has the same host-plant association (*Eriobotrya* and *Pyrus*) as *N*. *piri*^45^. From these aspects, we may assume that the host-plant selection of *Nippolachnus* spp. may not be completely random. However, it is not clear why each group only occurs on a specific host-plant pair (*Eriobotrya* and *Pyrus* or *Rhaphiolepis* and *Sorbus*).

Among the four different host-plant associated populations, *N*. *piri* on *Pyrus* and *N*. *micromeli* on *Rhaphiolepis* showed greatest the haplotype diversity. These results may suggest that the host-plant relationships of *N*. *piri* on *Pyrus* and *N*. *micromeli* on *Rhaphiolepis* may be a more ancient compared to *N*. *piri* on *Eriobtrya* and *N*. *micromeli* on *Sorbus*. However, to answer the avobe question, additional phylogenetic studies of *Nippolachnus* spp. are needed in the future.

The present study indicated that *Nippolachnus piri* is a species complex rather than a polyphagous and widely distributed species. Based on the combined morphological and molecular analyses, *Nippolachnus piri* on the four host-associated populations can be separated into the two distinct groups: A (on *Sorbus* and *Rhaphiolepis*) and B (on *Pyrus* and *Eriobotrya*) are characterised by variously pigmented and sclerotised apterous and alate viviparous females, which are similar in live and mounted specimens. After detailed analyses of previously described species, and with only the brief description of Shinji^15^, we believe that the representatives of group A should be treated as *N*. *micromeli* rather than a new, hitherto unknown species, while the specimens from the group B correspond with the host plant and morphology of Matsumura’s *N*. *piri*.

The apterous viviparous females of *N*. *piri* are characterised by a greenish body with green markings on the dorsal side of the body and brown hind tibiae with a dark distal part and dark hind tarsi. The same morphs in *N*. *micromeli* are almost completely pale or whitish with only the distal part of hind tibiae and tarsi being brown. Apterae of both species can also be distinguished by the pigmentation of the distal part of antennae, which in *N*. *piri* are brown in contrast to the pale or whitish antennae in *N*. *micromeli*. The pigmentation differences on the tibiae and antennae of apterous females are also manifested in macerated specimens. Alate viviparous females are characterised by more morphological differences, especially in the sclerotization of the dorsal and lateral side of the abdomen. In the alate viviparae of *N*. *piri*, the abdomen has a uniform, large spino-pleural cross-bar on ABD I and marginal sclerites on ABD I-IV. Additionally, the area between the pleural and marginal sclerites on ABD I-III and near SIPH is characterised by distinct sclerites at the setal bases. Abdominal segments VI and VII are free from sclerites or cross-bars. The alate in *N*. *micromeli* also differ from the latter by a broken or double spino-pleural bar on ABD I, marginal sclerites on only ABD II and III, lack of sclerites at setal bases and the presence of sclerites on ABD VI and VII. The morphs of both species also differ in the number of secondary rhinaria on ANT III. A detailed comparison between specific morphs of both species is presented in Table 1.

Shinji was one of the best known Japanese aphidologists in the first half of the 20^th^ century. Although he is known as the author of many aphid species from Japan, unfortunately, many of his descriptions were quite short and were based on live specimens without accounts on the detailed morphology and measurements of macerated specimens. Moreover, it is known that Shinji’s collection is has been lost. *N*. *micromeli* was never collected again after Shinji’s publication. The absence of any material has raised doubts about the identity or validity of some species. In the case of *N*. *micromeli*, an inaccurate and insufficient description together with the absence of any material was most probably the reason that it was treated as synonym of *N*. *piri* despite some significant differences.

Scanning electron microscopy showed that the species within the genus *Nippolachnus* are similar on this level and demonstrate similar features. The body shape is similar and is covered by quite long and very fine setae which in many cases may be trichoid sensilla, especially on the antennae and legs. The antennae have the greatest number of sensilla. On the pedicel, two rhinariola can be found, which lie on the more or less visible basal part and are similar to coeloconic sensilla. Moreover, a well-visible campaniform sensillum with a wide and rounded basal part is visible on the dorsal side of this segment. The trichoid sensilla on all of the antennal segments are similar to the others that cover the body and legs. One of the most characteristic features of the representatives of this genus are the enlarged placoid sensilla (big multiporous sensilla) – the primary and secondary rhinaria, especially in the alate viviparous females. Another characteristic is the arrangement of the accessory rhinaria (small placoid sensilla and coeloconic sensilla), which are almost totally shifted on the terminal process of the last antennal segment. This type of accessory sensilla arrangement is different than in the other lachnids that have been analysed to date. In the Eulachnini genus *Eulachnus*, the accessory sensilla are located under the major rhinarium (large placoid sensillum)^46^, while in *Pseudessigella* one (small placoid sensillum) is separated from the others and is located on the side of the major rhinarium^47^. Although only the arrangement of sensilla of *Tuberolachnus salignus* is so far known in the tribe Tuberolachnini, it is very different from that of *Nippolachnus* in which all of the accessory rhinaria are arranged linearly on the side of the major rhinarium, in all of the cases that are known, the accessory rhinaria lie on the basal part of the last antennal segment^48^.

## Methods

### Taxon sampling

A total of 41 individuals of *Nippolachnus piri* were collected in Korea (15 specimens on *Pyrus pyrifolia*, six specimens on *Rhaphiolepis indica*, 13 specimens on *Sorbus alnifolia*) and Japan (two specimens on *Eriobotrya japonica* and five specimens on *Rhaphiolepis indica*) (Fig. 1). Each colony of aphids was preserved in 95–99% ethanol at −20 °C to preserve DNA. The detailed collection information and Genbank accession numbers are listed in S Table 1.

Occurrence data were based on a detailed review of specimens that had been studied in museum collections and in the scientific literature. All of the localities were retrospectively georeferenced using Google Earth 7.1.2.2041^49^ – the coordinates were determined by describing the locality (geographical projection, decimal degrees and datum: WGS84). The localities of the host plants are from the Global Biodiversity Information Facility (GBIF; http://www.gbif.org/). Only those localities with precisely formulated coordinates were selected from among the sites of the occurrence of the host plants. Repetitions and imprecise data were deleted.

The map (S Fig. 1) was prepared in SAGA GIS 3.0.0 (http://www.saga-gis.org)^50^ using WGS84 datum and EPSG: 3395 (World Mercator).

### Material examined

*Nippolachnus piri* Matsumura, 1917.

As there is no information about whether any type material was designated during the original description and no material of Matsumura was available to the authors, no name-bearing type specimen is believed to be extant and the authors believe that a name-bearing type is necessary in order to define the nominal taxon objectively. According to the International Code of Zoological Nomenclature (Article 75.1), the Neotype of *Nippolachnus piri* is designated here:

**Neotype** (present designation), JAPAN: alate viviparous female (al.), Okitsu, Shizuoka-ken, 16.04.2015, *Eriobotrya japonica*, M. Sano leg., Jap15/04/1, UŚ.

Other material: KOREA: apterous viviparous female (apt.), Is. Odongdo, Sujeong-dong, Yeosu-si, Jeollanam-do, 14.07.2014, *Pyrus pyrifolia*, Y. Lee leg., Kor14/07/01, UŚ, two apt. Kor14/07/02, UŚ; one apt., Chusan experimental forest, Gwangyang-si, Jeollanam-do, 17.06.2016, *P*. *pyrifolia*, Y. Lee leg., Kor16/06/1, UŚ, one apt., one al., Kor16/06/2, UŚ, one apt., one al., Kor16/06/3, UŚ, one apt., one al., Kor16/06/4, UŚ;

More information about the material is presented in S Table 1.

*Nippolachnus micromeli* Shinji, 1924.

As there is no information that during the original description any type material was designated and no material of Shinji is available for the authors, no name-bearing type specimen is believed to be extant and the authors consider that a name-bearing type is necessary to define the nominal taxon objectively. According to the International Code of Zoological Nomenclature (Article 75.1), the Neotype of *Nippolachnus micromeli* is here designated:

**Neotype** (present designation): JAPAN: one al., Amakubo, Tsukuba-shi, Ibaraki-ken, 10.10.2016, *Rhaphiolepis mbellate*, M. Miyazaki leg., Jap16/10/2, UŚ.

**Other material:** JAPAN: one apt., Amakubo, Tsukuba-shi, Ibaraki-ken, 10.10.2016, *Rhaphiolepis umbellata*, M. Miyazaki leg., Jap15/10/1, UŚ; one al., Jap16/10/3, UŚ, one al., Jap16/10/4, UŚ, KOREA: one al., Mt. Oseo, Boryeong-gun, Chuncheongnam-do, 15.10.2011, *Sorbus alnifolia*, Y. Lee leg., Kor11/10/1 (SNU), one al., Kor11/10/2, UŚ; one al., Jeonnam Univ. arboretum, Bogil-myeon, Wando-gun, Jeollanam-do, 07.05.2016, *Rhaphiolepis umbellata*, Y. Lee leg., Kor16/05/1, UŚ, one al., Kor16/05/2, UŚ, two al., Kor16/05/3, UŚ, two al., Kor16/05/4, UŚ; two apt., Is. Geumohdo, Yeosu-si, Jeollanam-do, 05.06.2016, *Sorbus* sp., J. Choi leg., Kor16/06/1 UŚ, three apt., Kor16/06/2, UŚ.

More material information of remaining species of the genus *Nippolachnus* in S Table 1.

### Species identification

The specimens were mounted in Canada balsam following the method of Blackman and Eastop^51^ and Martin^52^ or in Faure-Berlese fluid following the method of Kanturski and Wieczorek^53^. The specimens were examined using a Nikon Eclipse e600 light microscope and photographed with a DS-Fi2 digital camera. The following abbreviations are used: BL – body length (from the anterior border of the head to the end of the cauda); HW – head width across the compound eyes; ANT – antennae or their length; ANT I-V – antennal segments I-V or their lengths (ratios between the antennal segments are simply given as e.g. ‘V:III’); LS III – length of the longest seta of ANT III; BD III – basal articular diameter of ANT III; BASE – basal part of the last antennal segment or its length; PT – processus terminalis of the last antennal segment or its length; URS – ultimate segment of the rostrum or its length; FEMORA III – hind femora length; TIBIAE III – hind tibiae length; HT – first segment of the hind tarsus or its length; HT II – second segment of the hind tarsus or its length and ABD I-VIII – abdominal tergites I-VIII.

The neotypes of *N*. *micromeli* and *N*. *piri* will be deposited at the Department of Zoology, University of Silesia (DZUS), Katowice, Poland. Specimens from the neotype series of both species will additionally be deposited at the Department of Zoology, University of Silesia (DZUS), Katowice, Poland; the Insect Museum, National Institute for Agro-Environmental Sciences, Tsukuba, Japan (NIAES) and the College of Agriculture and Life Sciences, Seoul National University, Seoul, Republic of Korea (CALS SNU).

### DNA extraction and DNA barcoding

 Whole genomic DNA was extracted from each sample that was selected from each colony using a DNeasy Blood & Tissue kit (Qiagen, Dusseldorf, Germany) according to modified manufacturer’s protocols. We used a non-destructive method^31^ to confirm the morphological features.

A 658 bp of the *Cytochrome oxidase I* gene (*COI*) was amplified using the universal primer sets: LCO1490 5′-GGTCAACAAATCATAAAGATATTGG-3′ and HCO2198 5′-TAAACTTCAGGGTGACCAAAAAATCA-3′^54^. A polymerase chain reaction (PCR) was performed with AccuPower PCR Premix (Bioneer, Daejeon, Rep. of Korea) in 20 ml reaction mixtures under the following conditions: initial denaturation at 94 °C for 3 min; followed by 35 cycles at 94 °C for 30 s, an annealing temperature of 45.2 °C for 30 s, an extension at 72 °C for 1 min and the final extension at 72 °C for 5 min. All PCR products were assessed using 1.5% agarose gel electrophoresis. Successfully amplified samples were purified using a QIAquick PCR purification kit (Qiagen, Inc.) and then sequenced immediately using an automated sequencer (ABI Prism 3730XL DNA Analyzer) at Bionics Inc. (Seoul, Korea).

### Molecular analyses

All sequences that were to be analysed were initially assembled and examined using Seqman pro ver. 7.1.0. (DNA star, Inc., Madison, Wisconsin, USA). Poor-quality sequences were discarded at this step in order to avoid errors. In total, 47 *COI* sequences of *Nippolachnus* spp. (41 that were produced in this study and six that were downloaded from Genbank) were aligned using the online utility MAFFT ver. 7 alignment package^55^ and MEGA 7^57^. Ambiguous anterior and posterior sequences were removed at this step. Lastly, 625 bp was used for the analyses. For the aligned dataset, a neighbour-joining analysis (NJ) was conducted using MEGA 7, which is based on the Kimura-2-Parameter (K2P) model^57^. *Pyrolachnus* sp. (Genbank accession number: KM501356) was used as an outgroup of the NJ analysis. Genetic distances between the groups were calculated using the pairwise distance method, which is based on the K2P model^53^ using MEGA 7. The *COI* haplotypes of four host-plant associated populations were analysed using DnaSP ver. 5.1.^58^. A median-joining network (MJ) was constructed using Network ver. 5.0.0.1.^59^.

### Scanning Electron Microscopy

Specimens for SEM analyses were preserved in 70% ethanol for several days. A method that was modified from Kanturski *et al*.^8^ was used to prepare the specimens. The specimens were transferred from the ethanol into a 6% phosphotungstic acid (PTA) solution in 70% ethanol for 24 hours. Dehydration was performed in an ethanol series of 80%, 90%, 96% and two changes of absolute ethanol for 30 minutes each. The dehydrated specimens were dried using a hexamethyldisilazane (HMDS) solution with absolute ethanol in proportions of 1:3, 1:2; 2:3 for 30 minutes each followed by two changes of undiluted HMDS. Samples were mounted on aluminium stubs using double-sided adhesive carbon tape and sputter-coated in a Pelco SC-6 sputter coater (Ted Pella Inc., Redding, CA, USA). The specimens were imaged using a Hitachi SU8010 field emission scanning electron microscope (FE-SEM) (Hitachi High-Technologies Corporation, Tokyo, Japan) at a 5, 10 and 15 kV accelerating voltage with a secondary electron detector (ESD).

## Electronic supplementary material

Dataset 1
