# Supplementary material for: DNA barcoding and a precise morphological comparison revealed a cryptic species in the Nippolachnus piri complex (Hemiptera: Aphididae: Lachninae)
Source: Sci Rep. 2018 Jun 13;8:8998. doi: 10.1038/s41598-018-27218-2 (PMC5997986; doi:10.1038/s41598-018-27218-2)

**DNA barcoding and a precise morphological comparison revealed a cryptic species in the *Nippolachnus piri* complex (Hemiptera: Aphididae: Lachninae)**

Mariusz Kanturski ^1*^, Yerim Lee ^2^, Jinyeong Choi ^2^, Seunghwan Lee ^2^,

^1^ Department of Zoology, Faculty of Biology and Environmental Protection, University of Silesia in Katowice, Bankowa 9, 40-007 Katowice, Poland.

^2^ Laboratory of Insect Biosystematics, Department of Agricultural Biotechnology, Research Institute of Agriculture and Life Sciences, Seoul National University, Seoul 08826, Republic of Korea

**SUPPLEMENTARY INFORMATION**

Scientific Reports

**Supplementary Table 1.** Detailed collection information and Genbank accession numbers of the species that were used in the molecular study and morphological comparison.

| **Genbank assesion no.** | **Voucher no.** | | **DNA no.** | | **Species** | | **Locality** | | **Date** | | **host plant** | | **Collector** |
| --- | --- | --- | --- | --- | --- | --- | --- | --- | --- | --- | --- | --- | --- |
| MG333573 | 150416MS-E2 | | Eri1-2 | | *Nippolachnus piri* | | JAPAN: Shizuoka-ken, Okitsu | | 16.iv.2015 | | *Eriobotrya japonica* | | Masakazu Sano |
| MG333574 | 150416MS-E3 | | Eri1-3 | | *Nippolachnus piri* | | JAPAN: Shizuoka-ken, Okitsu | | 16.iv.2015 | | *Eriobotrya japonica* | | Masakazu Sano |
| MG333575 | 140714YR-P1.1 | | Py1-1 | | *Nippolachnus piri* | | KOREA: JN, Yeosu-si, Is. Odongdo | | 14.vii.2014 | | *Pyrus pyrifolia* | | Yerim Lee |
| MG333576 | 140714YR-P1.2 | | Py1-2 | | *Nippolachnus piri* | | KOREA: JN, Yeosu-si, Is. Odongdo | | 14.vii.2014 | | *Pyrus pyrifolia* | | Yerim Lee |
| MG333577 | 140714YR-P1.3 | | Py1-3 | | *Nippolachnus piri* | | KOREA: JN, Yeosu-si, Is. Odongdo | | 14.vii.2014 | | *Pyrus pyrifolia* | | Yerim Lee |
| MG333578 | 140714YR-P1.4 | | Py1-4 | | *Nippolachnus piri* | | KOREA: JN, Yeosu-si, Is. Odongdo | | 14.vii.2014 | | *Pyrus pyrifolia* | | Yerim Lee |
| MG333579 | 140714YR-P1.5 | | Py1-5 | | *Nippolachnus piri* | | KOREA: JN, Yeosu-si, Is. Odongdo | | 14.vii.2014 | | *Pyrus pyrifolia* | | Yerim Lee |
| MG333580 | 140714YR-P1.6 | | Py1-6 | | *Nippolachnus piri* | | KOREA: JN, Yeosu-si, Is. Odongdo | | 14.vii.2014 | | *Pyrus pyrifolia* | | Yerim Lee |
| MG333581 | 140714YR-P1.7 | | Py1-7 | | *Nippolachnus piri* | | KOREA: JN, Yeosu-si, Is. Odongdo | | 14.vii.2014 | | *Pyrus pyrifolia* | | Yerim Lee |
| MG333582 | 140714YR-P1.8 | | Py1-8 | | *Nippolachnus piri* | | KOREA: JN, Yeosu-si, Is. Odongdo | | 14.vii.2014 | | *Pyrus pyrifolia* | | Yerim Lee |
| MG333583 | 160617YR-P2.2 | | Py2-2 | | *Nippolachnus piri* | | KOREA: JN, Gwangyang-si, Chusan-experimental forest | | 17.vi.2016 | | *Pyrus pyrifolia* | | Yerim Lee |
| MG333584 | 160617YR-P2.3 | | Py2-3 | | *Nippolachnus piri* | | KOREA: JN, Gwangyang-si, Chusan-experimental forest | | 17.vi.2016 | | *Pyrus pyrifolia* | | Yerim Lee |
| MG333585 | 160617YR-P2.4 | | Py2-4 | | *Nippolachnus piri* | | KOREA: JN, Gwangyang-si, Chusan-experimental forest | | 17.vi.2016 | | *Pyrus pyrifolia* | | Yerim Lee |
| MG333586 | 160617YR-P2.5 | | Py2-5 | | *Nippolachnus piri* | | KOREA: JN, Gwangyang-si, Chusan-experimental forest | | 17.vi.2016 | | *Pyrus pyrifolia* | | Yerim Lee |
| MG333587 | 160617YR-P2.6 | | Py2-6 | | *Nippolachnus piri* | | KOREA: JN, Gwangyang-si, Chusan-experimental forest | | 17.vi.2016 | | *Pyrus pyrifolia* | | Yerim Lee |
| MG333588 | 160617YR-P2.7 | | Py2-7 | | *Nippolachnus piri* | | KOREA: JN, Gwangyang-si, Chusan-experimental forest | | 17.vi.2016 | | *Pyrus pyrifolia* | | Yerim Lee |
| MG333589 | 160617YR-P2.8 | | Py2-8 | | *Nippolachnus piri* | | KOREA: JN, Gwangyang-si, Chusan-experimental forest | | 17.vi.2016 | | *Pyrus pyrifolia* | | Yerim Lee |
| MG333590 | 160507JY-R1.1 | | Rap1-1 | | *Nippolachnus* sp.1 ex. *Raphiolepis* | | KOREA: JN, Gwangju-si, Jeonnam National Univ. arboretum | | 7.v.2016 | | *Raphiolepis indica* | | Jinyeong Choi |
| MG333591 | 160507JY-R1.2 | | Rap1-2 | | *Nippolachnus* sp.1 ex. *Raphiolepis* | | KOREA: JN, Gwangju-si, Jeonnam National Univ. arboretum | | 7.v.2016 | | *Raphiolepis indica* | | Jinyeong Choi |
| MG333592 | 160507JY-R1.3 | | Rap1-3 | | *Nippolachnus* sp.1 ex. *Raphiolepis* | | KOREA: JN, Gwangju-si, Jeonnam National Univ. arboretum | | 7.v.2016 | | *Raphiolepis indica* | | Jinyeong Choi |
| MG333593 | 160507JY-R1.4 | | Rap1-4 | | *Nippolachnus* sp.1 ex. *Raphiolepis* | | KOREA: JN, Gwangju-si, Jeonnam National Univ. arboretum | | 7.v.2016 | | *Raphiolepis indica* | | Jinyeong Choi |
| MG333594 | 160507JY-R1.5 | | Rap1-5 | | *Nippolachnus* sp.1 ex. *Raphiolepis* | | KOREA: JN, Gwangju-si, Jeonnam National Univ. arboretum | | 7.v.2016 | | *Raphiolepis indica* | | Jinyeong Choi |
| MG333595 | 160507JY-R1.6 | | Rap1-6 | | *Nippolachnus* sp.1 ex. *Raphiolepis* | | KOREA: JN, Gwangju-si, Jeonnam National Univ. arboretum | | 7.v.2016 | | *Raphiolepis indica* | | Jinyeong Choi |
| MG333596 | 161010MM-RJP1.1 | | RapJP1-1 | | *Nippolachnus* sp.1 ex. *Raphiolepis* | | JAPAN: Ibaraki-ken, Tsukuba-shi, Amakubo | | 10.x.2016 | | *Rhaphiolepis umbellata* | | Masahisa Miyazaki |
| MG333597 | 161010MM-RJP1.2 | | RapJP1-2 | | *Nippolachnus* sp.1 ex. *Raphiolepis* | | JAPAN: Ibaraki-ken, Tsukuba-shi, Amakubo | | 10.x.2016 | | *Rhaphiolepis umbellata* | | Masahisa Miyazaki |
| MG333598 | 161010MM-RJP1.3 | | RapJP1-3 | | *Nippolachnus* sp.1 ex. *Raphiolepis* | | JAPAN: Ibaraki-ken, Tsukuba-shi, Amakubo | | 10.x.2016 | | *Rhaphiolepis umbellata* | | Masahisa Miyazaki |
| MG333599 | 161010MM-RJP1.4 | | RapJP1-4 | | *Nippolachnus* sp.1 ex. *Raphiolepis* | | JAPAN: Ibaraki-ken, Tsukuba-shi, Amakubo | | 10.x.2016 | | *Rhaphiolepis umbellata* | | Masahisa Miyazaki |
| MG333600 | 161010MM-RJP1.5 | | RapJP1-5 | | *Nippolachnus* sp.1 ex. *Raphiolepis* | | JAPAN: Ibaraki-ken, Tsukuba-shi, Amakubo | | 10.x.2016 | | *Rhaphiolepis umbellata* | | Masahisa Miyazaki |
| MG333601 | 160505JY-S1.1 | | Sor1-1 | | *Nippolachnus* sp.1 ex. *Sorbus* | | KOREA: JN, Yeosu-si, Is. Geumohdo | | 5.v.2016 | | *Sorbus alnifolia* | | Jinyeong Choi |
| MG333602 | 160505JY-S1.2 | | Sor1-2 | | *Nippolachnus* sp.1 ex. *Sorbus* | | KOREA: JN, Yeosu-si, Is. Geumohdo | | 5.v.2016 | | *Sorbus alnifolia* | | Jinyeong Choi |
| MG333603 | 160505JY-S1.4 | | Sor1-4 | | *Nippolachnus* sp.1 ex. *Sorbus* | | KOREA: JN, Yeosu-si, Is. Geumohdo | | 5.v.2016 | | *Sorbus alnifolia* | | Jinyeong Choi |
| MG333604 | 160505JY-S1.5 | | Sor1-5 | | *Nippolachnus* sp.1 ex. *Sorbus* | | KOREA: JN, Yeosu-si, Is. Geumohdo | | 5.v.2016 | | *Sorbus alnifolia* | | Jinyeong Choi |
| MG333605 | 160505JY-S1.6 | | Sor1-6 | | *Nippolachnus* sp.1 ex. *Sorbus* | | KOREA: JN, Yeosu-si, Is. Geumohdo | | 5.v.2016 | | *Sorbus alnifolia* | | Jinyeong Choi |
| MG333606 | 160505JY-S1.7 | | Sor1-7 | | *Nippolachnus* sp.1 ex. *Sorbus* | | KOREA: JN, Yeosu-si, Is. Geumohdo | | 5.v.2016 | | *Sorbus alnifolia* | | Jinyeong Choi |
| MG333607 | 160714HJ-SIJ2 | | Sor-IJ2 | | *Nippolachnus* sp.1 ex. *Sorbus* | | KOREA: GW, Inje-gun, Morangol | | 14.vii.2016 | | *Sorbus alnifolia* | | Huijun An |
| MG333608 | 160714HJ-SIJ3 | | Sor-IJ3 | | *Nippolachnus* sp.1 ex. *Sorbus* | | KOREA: GW, Inje-gun, Morangol | | 14.vii.2016 | | *Sorbus alnifolia* | | Huijun An |
| MG333609 | 160714HJ-SIJ4 | | Sor-IJ4 | | *Nippolachnus* sp.1 ex. *Sorbus* | | KOREA: GW, Inje-gun, Morangol | | 14.vii.2016 | | *Sorbus alnifolia* | | Huijun An |
| MG333610 | 160714HJ-SIJ5 | | Sor-IJ5 | | *Nippolachnus* sp.1 ex. *Sorbus* | | KOREA: GW, Inje-gun, Morangol | | 14.vii.2016 | | *Sorbus alnifolia* | | Huijun An |
| MG333611 | 140801YR-SJJ1 | | Sor-JJ1 | | *Nippolachnus* sp.1 ex. *Sorbus* | | KOREA: JJ, Jeju-si, Mt. Hallasan | | 1.viii.2014 | | *Sorbus alnifolia* | | Yerim Lee |
| MG333612 | 140801YR-SJJ2 | | Sor-JJ2 | | *Nippolachnus* sp.1 ex. *Sorbus* | | KOREA: JJ, Jeju-si, Mt. Hallasan | | 1.viii.2014 | | *Sorbus alnifolia* | | Yerim Lee |
| MG333613 | 140801YR-SJJ3 | | Sor-JJ3 | | *Nippolachnus* sp.1 ex. *Sorbus* | | KOREA: JJ, Jeju-si, Mt. Hallasan | | 1.viii.2014 | | *Sorbus alnifolia* | | Yerim Lee |
|  |  | |  | |  | |  | |  | |  | |  |
| Microscopic slides of *Nippolachnus* species used for morphological comparison and key construction: | | | | | | | | | | | | | |
| **Species name** | | **Slide no.** | | **Collection** | | **Locality and morph** | | **Date** | | **Host plant** | | **Collector** | |
| *N. himalayensis*  paratypes | | BM 1984-340 | | BMNH | | India, Darjeeling, one apt. viv. fem. | | 2.vi.1957 | | *Eriobotrya petiolata* | | A. N. Basu | |
|  |  | BM 1984-340 | | BMNH | | India, Darjeeling, one apt. viv. fem. | | 2.vi.1957 | | *Eriobotrya petiolata* | | A. N. Basu | |
|  |  | BM 1984-340 | | BMNH | | India, Darjeeling, one apt. viv. fem. | | 2.vi.1957 | | *Eriobotrya petiolata* | | A. N. Basu | |
| *N. bengalensis*  paratypes | | BM 1984-340 | | BMNH | | India, Darjeeling, two apt. and one al. viv. fem. | | 26.v.1958 | | *Eriobotrya petiolata* | | S. Das | |
| *N. bengalensis* | | BM 1984-340 | | BMNH | | India, Darjeeling, two apt. viv. fem. | | 15.xi.1968 | | *Eriobotrya dubia* | | S.G. Rajasingh | |
|  |  | BM 1984-340 | | BMNH | | India, Darjeeling, two apt. viv. fem. | | 6.i.1958 | | *Eriobotrya dubia* | | S. Das | |
| *N. xitianmushanus* | | BM 2004-145 | | BMNH | | China, Zhejiang Tianmushan, two. al. viv. fem. | | 13.v.1975 | | *Eriobotrya* sp.? | | G. X. Zhang | |

**Supplementary Table 2**. Measurements of *Nippolachnus piri* and *N. micromeli* stat. rev.

| **Character** | ***Nippolachnus piri*** | | ***Nippolachnus micromeli*** | |
| --- | --- | --- | --- | --- |
|  | **Apt. viv. fem.** | **Al. viv. fem.** | **Apt. viv. fem.** | **Al. viv. fem.** |
| **BL** | 2.27-3.55 | 2.90-4.05 | 2.25-2.97 | 2.87-3.25 |
| **HW** | 0.50-0.59 | 0.55-0.70 | 0.44-0.50 | 0.56-0.64 |
| **ANT** | 0.91-1.03 | 0.92-1.16 | 0.75-0.84 | 0.98-1.04 |
| **ANT III** | 0.30-0.34 | 0.31-0.39 | 0.24-0.29 | 0.32-0.38 |
| **ANT IV** | 0.10-0.14 | 0.11-0.15 | 0.07-0.10 | 0.12-0.14 |
| **ANT V** | 0.16-0.19 | 0.16-0.19 | 0.13-0.15 | 0.18-0.21 |
| **ANT VI** | 0.17-0.20 | 0.18-0.23 | 0.14-0.18 | 0.19-0.20 |
| **BASE** | 0.11-0.13 | 0.12-0.16 | 0.09-0.12 | 0.13 |
| **PT** | 0.05-0.07 | 0.05-0.07 | 0.05-0.06 | 0.06-0.07 |
| **URS** | 0.17-0.20 | 0.17-0.19 | 0.13-0.15 | 0.15-0.17 |
| **III FEMUR** | 1.20-1.60 | 1.37-1.75 | 0.90-1.12 | 1.37-1.60 |
| **III TIBIA** | 2.37-0.82 | 2.60-3.20 | 1.70-2.15 | 2.50-2.82 |
| **HT I b.** | 0.04 | 0.03-0.04 | 0.03 | 0.03 |
| **HT I d.** | 0.02 | 0.01-0.02 | 0.01 | 0.02 |
| **HT I v.** | 0.07-0.09 | 0.07-0.10 | 0.07-0.08 | 0.08-0.09 |
| **HT I i.** | 0.05-0.07 | 0.05-0.07 | 0.05-0.06 | 0.05-0.06 |
| **HT II** | 0.22-0.25 | 0.21-0.25 | 0.19-0.22 | 0.22-0.24 |
| **SIPH sclerite** | 0.28-0.37 | 0.34-0.50 | 0.25-0.30 | 0.32-0.45 |
| **SIPH pore** | 0.12-0.13 | 0.11-0.14 | 0.08-0.10 | 0.09-0.12 |
| **Fore wings L** | - | 3.45-5.00 | - | 4.55-5.25 |
| **GPL** | 0.10-0.16 | 0.17-0.26 | 0.10-0.15 | 0.16-0.21 |
| **GPW** | 0.16-0.30 | 0.32-0.41 | 0.20-0.25 | 0.26-0.34 |

**Supplementary Figure 1**. Distribution of the collection localities of the specimens of *Nippolachnus piri* species complex that were analysed in this study.


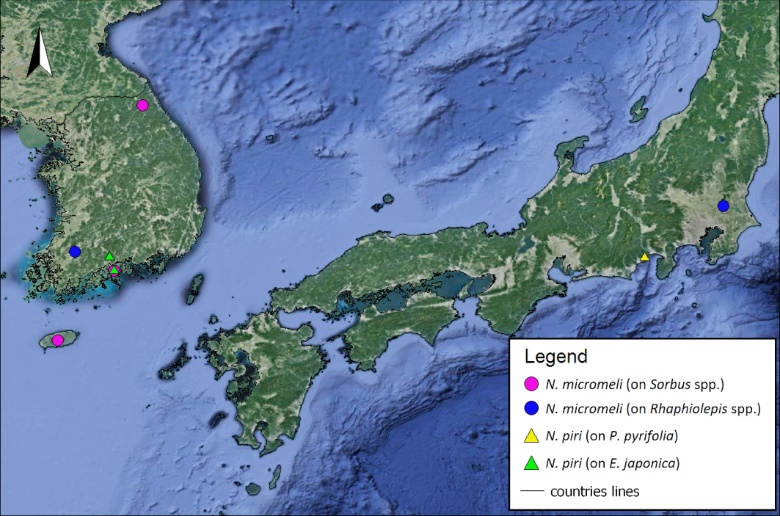


**Supplementary Figure 2**. Comparison of the antennae and hind legs in *Nippolachnus piri* complex: (**a**) ANT of *N. piri*; (**b**) ANT of *N. micromeli*; (**c**) hind leg of *N. piri* with yellow tibiae; (**d**) hind leg of *N. micromeli*, with pale tibiae.


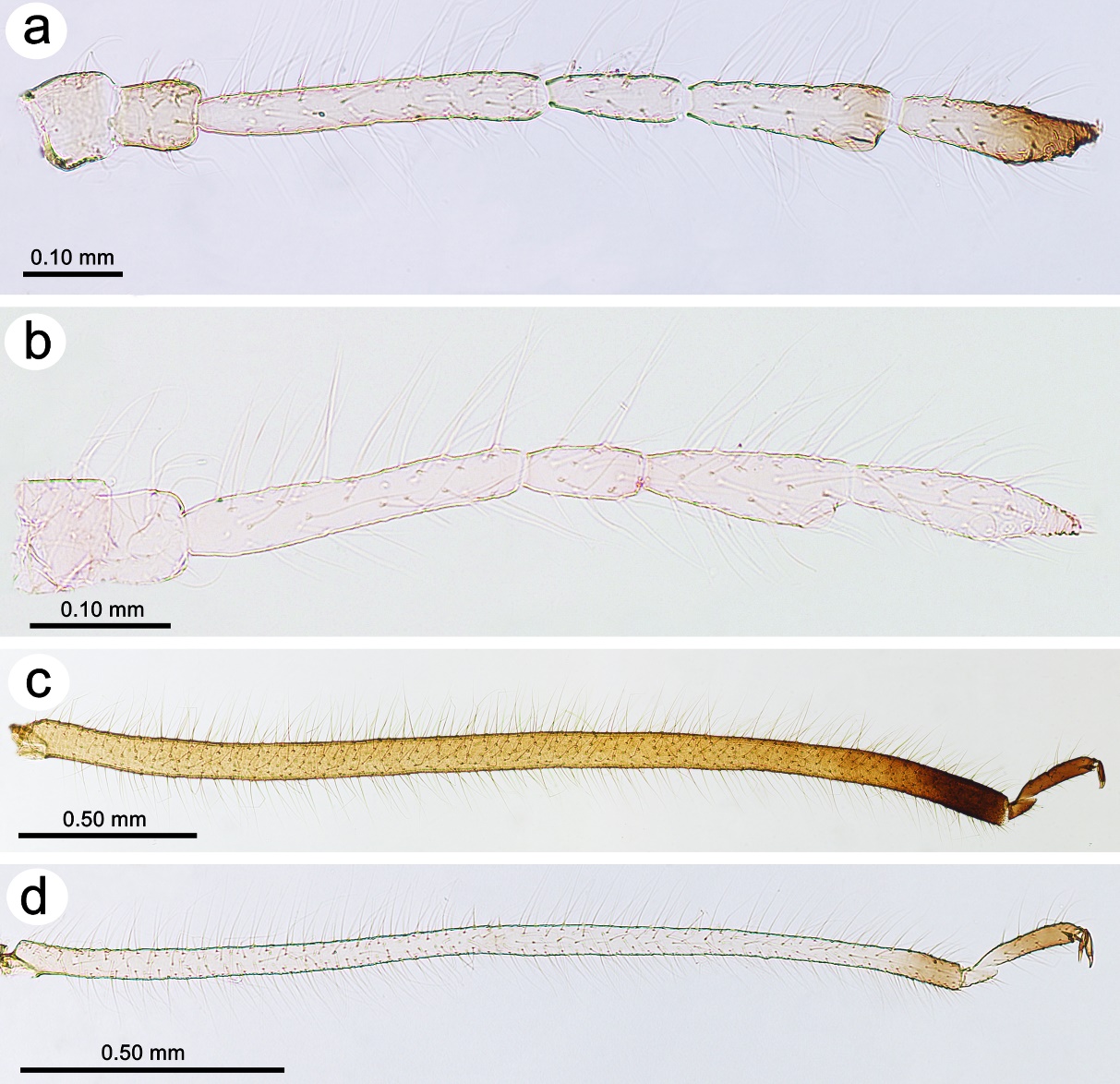


**Supplementary Figure 3**. Comparison of the antennae and hind legs in *Nippolachnus piri* complex: (**a**) ANT VI of *N. piri* with brown apex; (**b**) pale ANT VI of *N. micromeli*; (**c**) apical part of hind leg of *N. piri* with the dark brown end of the tibia and tarsus; (**d**) hind leg of *N. micromeli*, with only the yellow end of the tibia and the distal part of HT II.


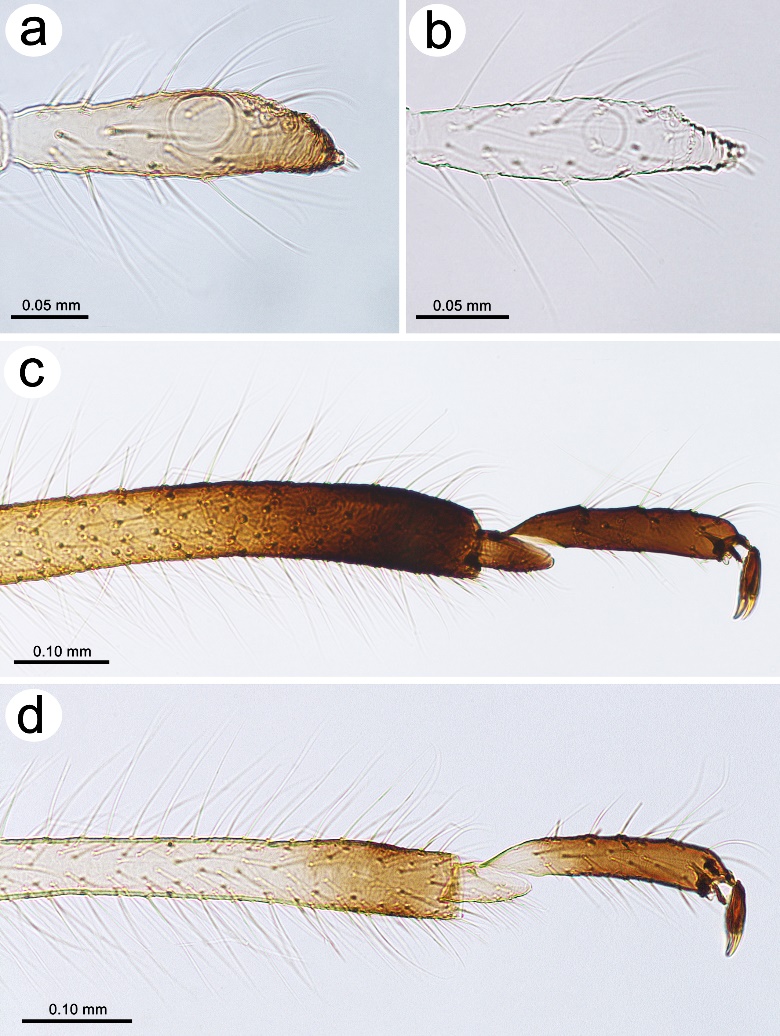


**Supplementary Figure 4**. Characters of an alate viviparous female of *N. piri*: (a) antenna; (b) ANT VI; (c) dorsal abdominal sclerotisation forming polygonal reticulation; (d) forewing; I-VI – antennal segments I-VI, white arrowhead – major rhinarium, black arrowhead – accessory rhinaria, Pt – pterostigma, Rs – radial sector, M, M_1_, M_2_ – media, Cu_1a_, Cu_1b_ – cubitus, black arrow on the wing shows the moment of the separation of the media relative to Rs and Pt.


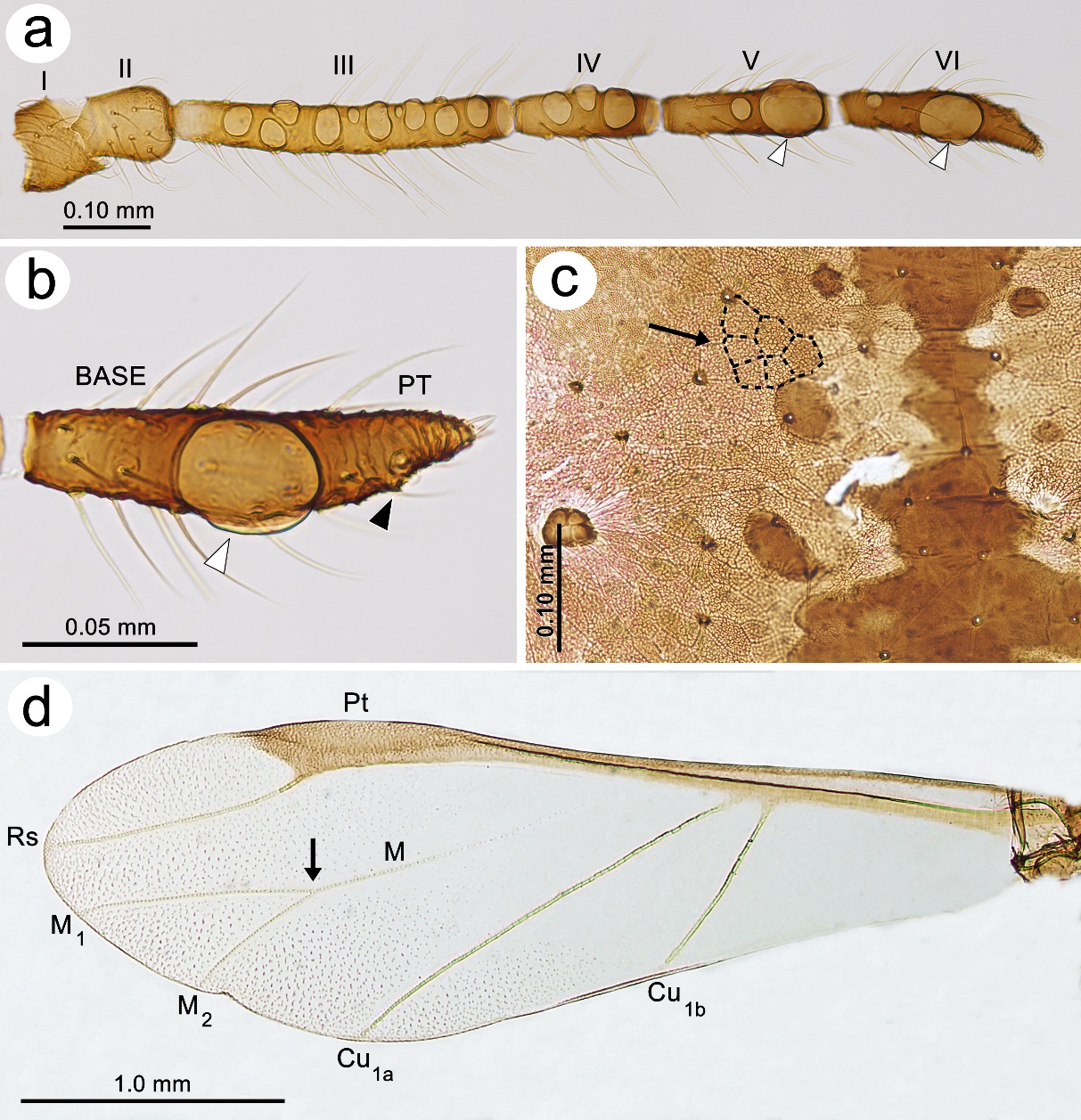


**Supplementary Figure 5**. Characters of an alate viviparous female of *N. micromeli*: (a) antenna; (b) ANT VI; (c) dorsal abdominal sclerotisation without polygonal reticulation; (d) forewing; I-VI – antennal segments I-VI, white arrowhead – major rhinarium, black arrowhead – accessory rhinaria, Pt – pterostigma, Rs – radial sector, M, M_1_, M_2_ – media, Cu_1a_, Cu_1b_ – cubitus, black arrow on the wing shows the moment of the separation of the media relative to Rs and Pt.


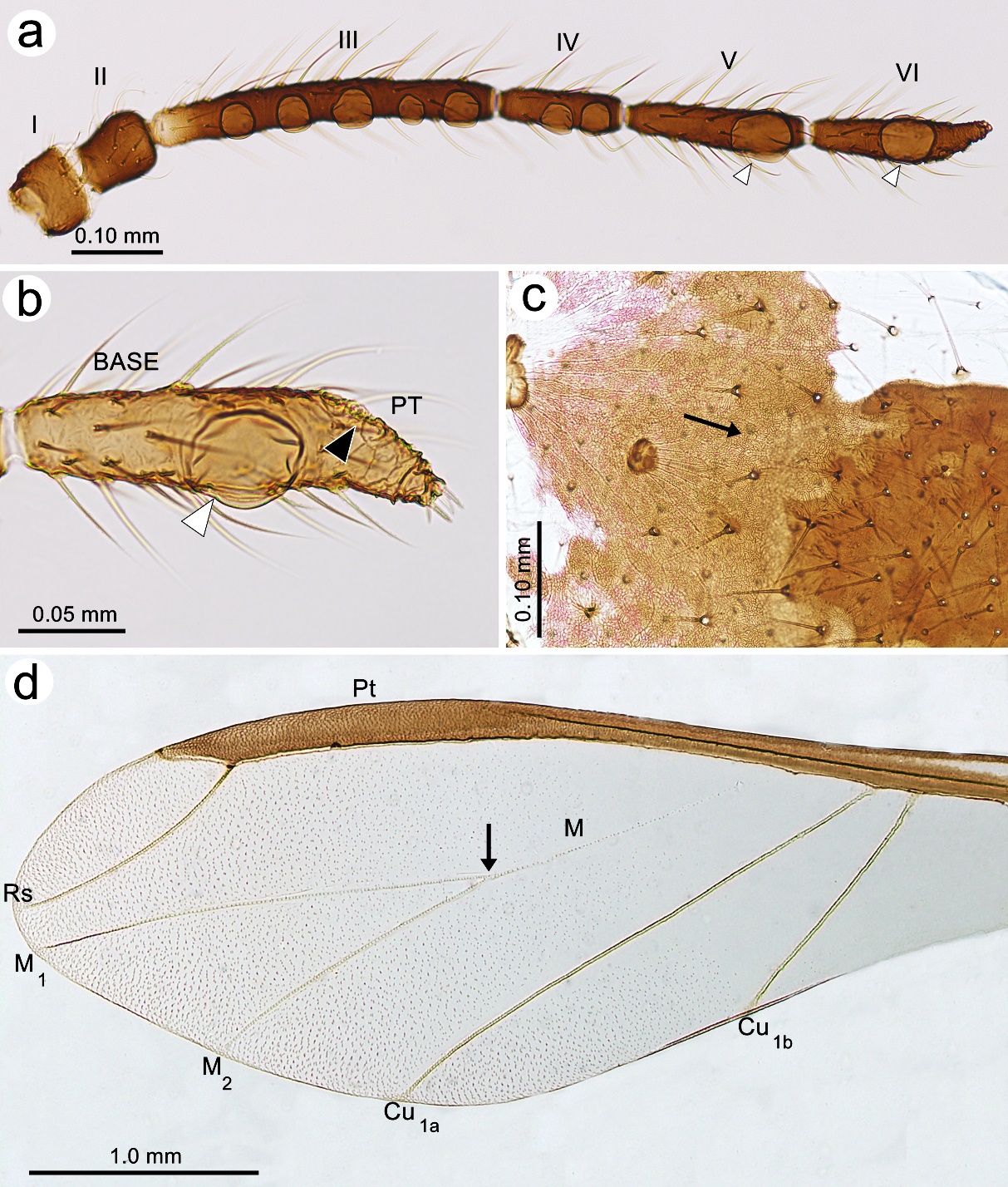


**Supplementary Figure 6**. SEM of the forewings of *Nippolachnus* representatives: (**a**) *N. piri*; (**b**) *N. micromeli* Pt – pterostigma, Rs – radial sector, M, M_1_, M_2_ – media, Cu_1a_, Cu_1b_ – cubitus.


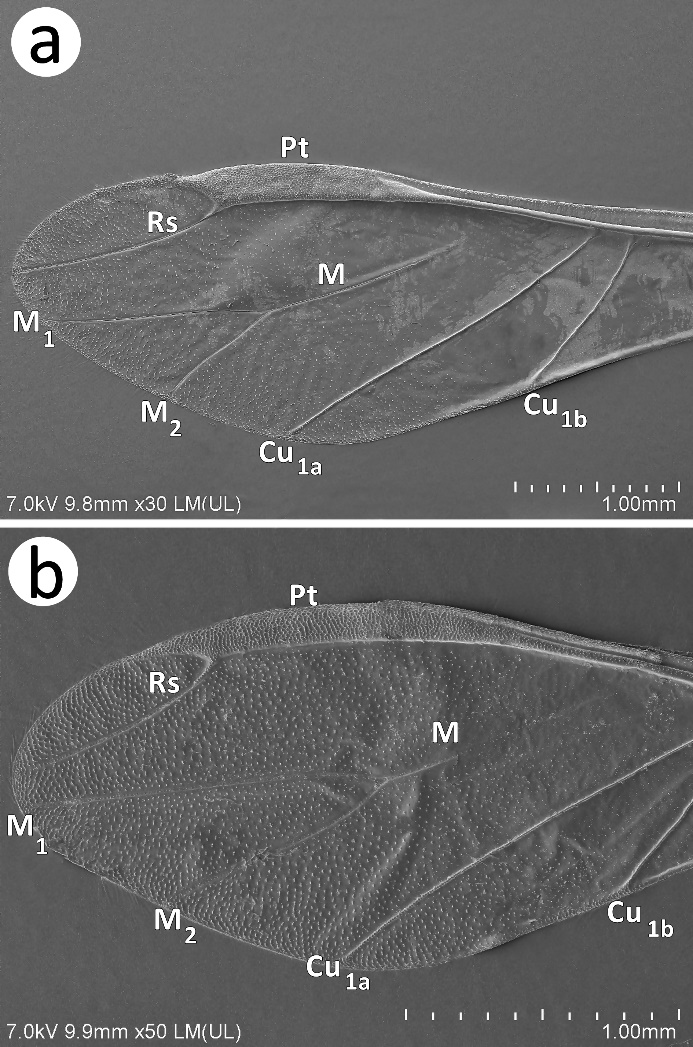

Supplement: Supplementary file 1 — Dataset 1 [file 41598_2018_27218_MOESM1_ESM.doc]
